# Community-based Malaria Screening and Treatment for Pregnant Women Receiving Standard Intermittent Preventive Treatment With Sulfadoxine-Pyrimethamine: A Multicenter (The Gambia, Burkina Faso, and Benin) Cluster-randomized Controlled Trial

**DOI:** 10.1093/cid/ciy522

**Published:** 2018-06-29

**Authors:** COSMIC Consortium, Susana Scott, Umberto D’Alessandro, Lindsay Kendall, John Bradley, Kalifa Bojang, Simon Correa, Fanta Njie, Halidou Tinto, Maminata Traore-Coulibaly, Hamtandi Magloire Natama, Ousmane Traoré, Innocent Valea, Alain Nahum, Daniel Ahounou, Francis Bohissou, Gethaime Sondjo, Carine Agbowai, Petra Mens, Esmée Ruizendaal, Henk Schallig, Susan Dierickx, Koen Peeters Grietens, Laetitia Duval, Lesong Conteh, Maxime Drabo, Jamie Guth, Franco Pagnoni

**Keywords:** malaria, pregnancy, sulfadoxine-pyrimethamine, artemether-lumefantrine, community-based malaria screening

## Abstract

**Background:**

We investigated whether adding community scheduled malaria screening and treatment (CSST) with artemether-lumefantrine by community health workers (CHWs) to standard intermittent preventive treatment in pregnancy with sulfadoxine-pyrimethamine (IPTp-SP) would improve maternal and infant health.

**Methods:**

In this 2-arm cluster-randomized, controlled trial, villages in Burkina Faso, The Gambia, and Benin were randomized to receive CSST plus IPTp-SP or IPTp-SP alone. CHWs in the intervention arm performed monthly CSST during pregnancy. At each contact, filter paper and blood slides were collected, and at delivery, a placental biopsy was collected. Primary and secondary endpoints were the prevalence of placental malaria, maternal anemia, maternal peripheral infection, low birth weight, antenatal clinic (ANC) attendance, and IPTp-SP coverage.

**Results:**

Malaria infection was detected at least once for 3.8% women in The Gambia, 16.9% in Benin, and 31.6% in Burkina Faso. There was no difference between study arms in terms of placenta malaria after adjusting for birth season, parity, and IPTp-SP doses (adjusted odds ratio, 1.06 [95% confidence interval, .78–1.44]; *P* = .72). No difference between the study arms was found for peripheral maternal infection, anemia, and adverse pregnancy outcomes. ANC attendance was significantly higher in the intervention arm in Burkina Faso but not in The Gambia and Benin. Increasing number of IPTp-SP doses was associated with a significantly lower risk of placenta malaria, anemia at delivery, and low birth weight.

**Conclusions:**

Adding CSST to existing IPTp-SP strategies did not reduce malaria in pregnancy. Increasing the number of IPTp-SP doses given during pregnancy is a priority.

**Clinical Trials Registration:**

NCT01941264; ISRCTN37259296.

## Teaser

Adding community-based scheduled malaria screening and treatment to existing intermittent preventive treatment with sulfadoxine-pyrimethamine (IPTp-SP) strategies did not reduce malaria in pregnancy. Increasing the number of IPTp-SP doses given during pregnancy continues to be a priority.

Malaria causes significant adverse pregnancy outcomes, such as maternal anemia, preterm delivery, low birth weight [1], and even maternal and infant death [2, 3]. The World Health Organization (WHO) recommends several interventions to control malaria during pregnancy, namely effective case management, long-lasting insecticidal nets, and intermittent preventive treatment with sulfadoxine-pyrimethamine (IPTp-SP) from the second trimester onward [4].

The protective efficacy of IPTp-SP against malaria infection depends on the number of IPTp-SP doses administered, which in turn depends on antenatal clinic (ANC) attendance. In many sub-Saharan African countries, both ANC and IPTp-SP coverage remains low [5, 6]. In addition, sulfadoxine-pyrimethamine (SP) resistance is increasing and may have an impact on current IPTp-SP policy [7]. In West Africa, where SP resistance is low [8], an alternative strategy of intermittent screening and treatment in pregnancy (ISTp) was noninferior to IPTp-SP in preventing low birth weight, anemia, and placental malaria. In southeast Africa, it was associated with a higher malaria risk, possibly because of the low sensitivity of currently available rapid diagnostic tests (RDTs) in detecting low-density infections [9, 10]. WHO does not recommend ISTp alone. However, there is the need to both improve ANC coverage and protect pregnant women against malaria between ANC visits. ISTp at the village level could be beneficial if given in addition to IPTp-SP at ANC. Community health workers (CHWs) have been trained in many sub-Saharan African countries to perform community case management of malaria, and could be trained to encourage pregnant women to attend the ANC and to systematically screen and treat them between ANC visits [11].

A cluster-randomized controlled trial was designed to establish whether adding community scheduled malaria screening and treatment (CSST) by CHWs to standard IPTp-SP would further reduce placental malaria compared to IPTp-SP alone [12].

## METHODS

### Study Sites and Participants

A description of the study methods has been published elsewhere [12]. The study was implemented in 3 West African countries: Burkina Faso (Nanoro health district), The Gambia (Upper River region), and Benin (Glo-Djigbe, Zinvie, and Ze districts). In Burkina Faso and The Gambia, malaria is highly seasonal (July–December), whereas in Benin it is perennial with peaks during the rainy seasons (April–July and October–November). In all villages, community consent was obtained after sensitization meetings. All resident pregnant women were invited to participate after individual signed informed consent.

### Randomization and Blinding

Thirty villages (clusters) (village population: 1000–2000) with CHWs in each country were randomly selected from all eligible clusters. Distance from the center of each village to the nearest health facility was calculated and used to group the clusters into 3 distance categories. Randomization was performed using computer-based randomization (Stata software, StataCorp, College Station, Texas) and was stratified by the 3 distance categories.

### Procedures

#### At the Antenatal Clinic

**Figure 1.**
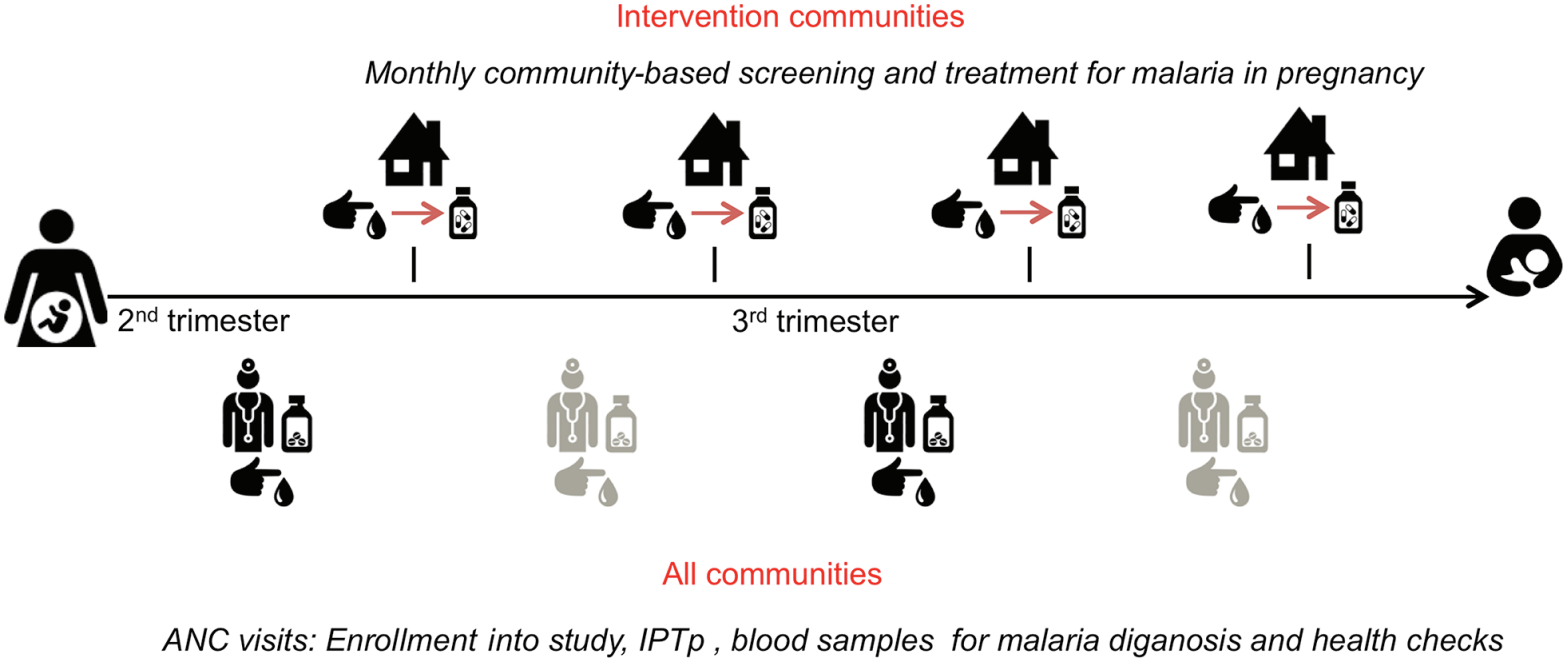
Schedule of events, updated from the Trial protocol paper [12]. Abbreviations: ANC, antenatal clinic; IPTp, intermittent preventive treatment in pregnancy.

Recruitment was done at first ANC (Figure 1). All women had a physical examination, a blood slide, and a blood sample on filter paper. Information on health and socioeconomic factors was also collected. Pregnant women in the second or third trimester were given their first IPTp-SP and their second dose booked in. A health assessment was carried out at each ANC visit. Suspected malaria cases had an RDT (SD Bioline; specificity 99.5%) and women testing positive were treated with artemether-lumefantrine (AL).

#### Community Health Worker Home Visits in the Intervention Arm

CHWs in the intervention arm were trained in malaria case management and malaria in pregnancy, including the benefit of early ANC attendance and IPTp-SP. CHWs were asked to continuously identify all pregnant women and encourage them to attend the ANC as early as possible. Thereafter, at monthly intervals up to the last week of gestation, CHWs performed an RDT at home visits and collected a blood slide, regardless of malaria symptoms. They gave AL to all positive women. Severely ill women were referred to the health center for further care. The CHWs in the control villages did not take part in any of the study training.

#### At Time of Delivery for All Communities

A blood sample for hemoglobin measurement and on slide and filter paper for later parasitological diagnosis was collected just before delivery, and a placenta biopsy was collected at delivery. Current health status and birth outcomes were collected. All newborns were physically examined and weighed on digital scales immediately after delivery. Gestational age was estimated using the Ballard score [13].

#### Laboratory Methods

Giemsa-stained thick blood films were read by 2 experienced microscopists, with discrepancies resolved by a third one [12]. Maternal hemoglobin was measured using Hb301 Hemocue (Radiometer Group, Sweden). Blood spots on filter paper were analyzed by *Plasmodium falciparum* diagnostic polymerase chain reaction (PCR) [14, 15].

Details of biopsy methods can be found elsewhere [12]. Placental biopsy slides were read by trained microscopists and classified as infected (acute: parasites and malaria pigment absent; chronic: parasites and malaria pigment; or past: only malaria pigment) or not infected (no parasites or pigment) [16].

### Outcomes

The primary outcome was placental malaria (any category). However, in Benin, rumors on placental biopsies had a negative impact on recruitment and, after discussions with the data and safety monitoring board (DSMB) and the local ethics committee, it was decided to collect only peripheral blood. Secondary endpoints were maternal anemia (hemoglobin <11 g/dL) at delivery, maternal *P. falciparum* peripheral infection at delivery (PCR) and during pregnancy (microscopy), low birth weight (<2500 g), IPTp-SP coverage, the number of ANC visits, and the number of IPTp-SP doses. Serious adverse events (SAEs) were defined as any untoward medical occurrence that resulted in death, hospitalization, persistent or significant disability/incapacity, or congenital anomaly/birth defect or that was life-threatening. All SAEs were reported to the DSMB.

### Statistical Analysis

It was assumed that the intervention would decrease placental malaria from 15% to 10.5%, with a coefficient of variation of 0.15. Within each country, 15 clusters, each with 60 pregnant women per arm, would be able to detect a significant difference with 80% power and at the 5% significance level. Thus, 90 clusters with a total of 5400 women were required. All data were double entered using OpenClinica databases.

The primary endpoint (prevalence of placental malaria) was examined with logistic regression. Random effects for trial cluster were used to account for intracluster correlation. An analysis of the primary endpoint adjusted for season, gravidity, and number of SP and AL doses was carried out. All other binary endpoints were examined with random effects logistic regression. Count data were analyzed with mixed effects Poisson regression and continuous data were analyzed with mixed effects linear regression.

### Ethical Approval

The study was done in accordance with the principles set forth in the Declaration of Helsinki and the International Conference on Harmonisation Tripartite Guidelines for Good Clinical Practice. Independent trial monitors visited each site throughout the study to ensure compliance with Good Clinical Practice standards. The trial was approved by the Gambia Government/Medical Research Council Joint Ethics Committee (reference number SCC1336), the Comité d’Ethique Institutionnel du Centre Muraz in Burkina Faso (reference number A20-2013/CE-CM), and the Comité National d’Ethique pour la Recherche en Santé in Benin (reference number 0126/MS /DC/SGM/DFR/CNERS/SA). A DSMB to review the trial procedures and results was set up. The trial is registered at Current Controlled Trials: ISRCTN37259296 (5 July 2013), and ClinicalTrials.gov: NCT01941264 (10 September 2013).

## RESULTS

### Recruitment, Baseline Characteristics, and Follow-up

**Figure 2.**
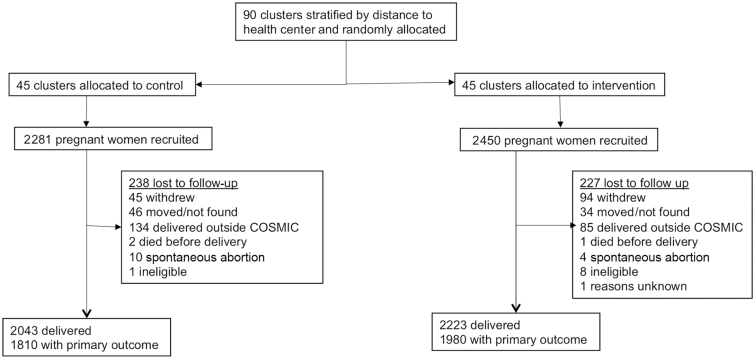
Flowchart of the cohort of pregnant women enrolled in the study. Primary outcome is placental malaria for the Burkina Faso and The Gambia study sites and maternal peripheral infection at time of delivery for the Benin study site.

Between November 2013 and November 2015, 4731 pregnant women were recruited (Figure 2 and Supplementary Figure 1*A–C*), with Benin having recruited only half of the expected sample size. Loss to follow-up was low in The Gambia (113/1960 [5.8%]) and in Burkina Faso (62/1800 [3.4%]) but high in Benin (290/971 [29.9%]). Overall, 4266 (90.2%) women completed the follow-up and delivered in study (Figure 2).

In each country, baseline characteristics between intervention and control groups were similar. Use of insecticide-treated bed nets was >70%, and malaria prevalence was 5.3% in The Gambia (101/1888), 27.9% in Burkina Faso (501/1797), and 30.0% in Benin (288/959). About 20% were primigravidae (Table 1).

**Table 1.** Baseline Characteristics of Study Clusters at Start of the Trial, by Country

Characteristic	Burkina Faso				Gambia				Benin			
	Control	%	Intervention	%	Control	%	Intervention	%	Control	%	Intervention	%
No. of clusters	15	…	15	…	15	…	15	…	15	…	15	…
Median No. of women per cluster (IQR)	60	(60–60)	60	(60–60)	69	(61–98)	67	(53–95)	41	(19–55)	48	(27–62)
Median age, y (IQR)	25	(20–30)	26	(21–30)	25	(20–29)	25	(20–29.5)	25	(21–29.5)	25	(20–30)
Ethnic group												
Mandinka	…	…	…	…	238	(25.0)	332	(32.9)	…	…	…	…
Fula	…	…	…	…	199	(20.9)	98	(9.7)	…	…	…	…
Serahuleh	…	…	…	…	510	(53.6)	577	(57.2)	…	…	…	…
Mossi	854	(94.9)	794	(88.2)	…	…	…	…	…	…	…	…
Aizo/Ouemenou	…	…	…	…	…	…	…	…	389	(90.7)	473	(87.27)
Other	46	(5.1)	106	(11.8)	5	(0.5)	1	(0.1)	40	(9.3)	69	(12.73)
Median gestational age, wk, at time of recruitment (IQR)	22	(20–24)	22	(20–24)	20	(17–22)	20	(17–22)	20	(18–24)	20	(18–24)
No. of previous pregnancies												
0	194	(21.6)	185	(20.6)	162	(17.1)	226	(22.4)	90	(21.0)	117	(21.6)
1	153	(17.0)	136	(15.1)	175	(18.4)	172	(17.1)	84	(19.6)	104	(19.2)
2	169	(18.8)	145	(16.1)	144	(15.2)	136	(13.5)	67	(15.6)	92	(17.0)
3	136	(15.1)	141	(15.7)	142	(15.0)	143	(14.2)	70	(16.3)	83	(15.3)
≥4	248	(27.6)	293	(32.6)	327	(34.4)	330	(32.8)	118	(27.5)	146	(26.9)
Marital status												
Married	759	(86.0)	832	(94.7)	930	(97.7)	987	(98.0)	206	(48.0)	245	(45.2)
Not married	124	(14.0)	47	(5.4)	22	(2.3)	20	(2.0)	223	(52.0)	297	(54.8)
Religion												
Christianity	521	(58.0)	466	(52.0)	…	…	…	…	386	(90.0)	499	(92.1)
Islam	215	(23.9)	306	(34.2)	935	(98.2)	986	(97.9)	…	…	…	…
Traditional African religion	134	(14.9)	111	(12.4)	…	…	…	…	…	…	…	…
Other	28	(4.9)	13	(1.5)	17	(1.8)	21	(2.1)	43	(10.0)	43	(7.9)
Woman’s occupation												
Housewife	818	(91.4)	802	(90.3)	109	(11.5)	122	(12.2)	114	(26.6)	156	(29.1)
Farmer/herder/gardener	47	(5.3)	67	(7.6)	719	(75.8)	760	(75.7)	42	(9.8)	46	(8.6)
Sell at market/shopkeeper	…	…	…	…	62	(6.5)	65	(6.5)	144	(33.6)	151	(28.1)
Childcare/domestic helper	…	…	…	…	45	(4.7)	50	(5.0)	…	…	…	…
Seamstress	…	…	…	…	…	…	…	…	65	(15.2)	95	(17.7)
Hairdresser	…	…	…	…	…	…	…	…	44	(10.3)	57	(10.6)
Other	30	(3.4)	19	(2.1)	14	(1.5)	7	(0.7)	19	(4.4)	32	(6.0)
Husband’s occupation												
Farmer/herdsman/fisherman/ gardener	707	(93.3)	789	(94.8)	578	(63.2)	591	(61.2)	103	(50.2)	103	(42.2)
Bricklayer/carpenter/welder	…	…	…	…	36	(3.9)	46	(4.8)	20	(9.8)	28	(11.5)
Sell at market/shopkeeper	…	…	…	…	84	(9.2)	66	(6.8)	…	…	…	…
Imam/marabout/VHW	…	…	…	…	23	(2.5)	20	(2.1)	…	…	…	…
Other	51	(6.7)	43	(5.2)	73	(8.0)	118	(12.2)	61	(29.8)	80	(32.8)
Traveler	…	…	…	…	105	(11.5)	107	(11.1)	…	…	…	…
Driver/motorbike driver	…	…	…	…	…	…	…	…	21	(10.2)	33	(13.5)
Teacher	…	…	…	…	15	(1.6)	17	(1.8)	…	…	…	…
Slept under treated net last night												
Yes	657	(73.2)	706	(78.7)	755	(80.2)	726	(72.9)	312	(72.7)	406	(74.9)
No	158	(17.6)	125	(13.9)	114	(12.1)	126	(12.7)	84	(19.6)	103	(19.0)
Do not have one	76	(8.5)	61	(6.8)	73	(7.6)	144	(14.5)	33	(7.7)	33	(6.1)
Don’t know if have one	6	(0.7)	5	(0.6)	…	…	…	…	…	…	…	…
Malaria positive by microscopy at recruitment												
No	640	(71.3)	656	(73.0)	870	(95.2)	917	(94.2)	300	(71.3)	371	(69.0)
Yes	258	(28.7)	243	(27.0)	44	(4.8)	57	(5.9)	121	(28.7)	167	(31.0)

Data are presented as No. (%) unless otherwise indicated.

Abbreviations: IQR, interquartile range; VHW, village health workers.

Most women in the intervention arm had at least 1 CSST by CHWs, with coverage higher in Burkina Faso (860/900 [96%]) and The Gambia (978/1008 [97%]) compared with Benin (474/542 [87.5%]). Overall, CHWs performed 7236 CSSTs, with an average of 3–4 home visits per woman (Supplementary Table 1). The mean number of slides per woman was significantly higher in the intervention than in the control group (Burkina Faso: means ratio [MR], 1.62 [95% confidence interval {CI}, 1.50–1.74]; The Gambia: MR, 2.08 [95% CI, 1.98–2.19]; Benin: MR, 2.04 [95% CI, 1.87–2.23]) (*P* < .001). Malaria infection was detected by RDT at least once for 75 (3.8%) women in The Gambia, 164 (16.9%) in Benin, and 568 (31.6%) in Burkina Faso, and most received AL (Table 2 and Supplementary Table 1). The number of pregnant women diagnosed with a malaria infection was significantly higher in the intervention arm than in the control arm (*P* < .01 for all study sites). A high proportion of infected women received AL, with no difference between study arms in Burkina Faso and The Gambia. In Benin, the proportion of treated women was higher in the intervention arm (172/191 [90.0%]) than in the control arm (14/33 [42.4%]) (odds ratio [OR], 1.71 [95% CI, .99–2.94]; *P* = .055).

**Table 2.** Total Number of Malaria Cases Diagnosed at Home Visits (Intervention Arm Only) and Antenatal Clinic Visits (Both Arms) by Rapid Diagnostic Test and Total Artemether-Lumefantrine Treatments Given During Study for All Countries

Cases	Overall		Control		Intervention		OR/RR	95% CI	*P* Value
	No.	%	No.	%	No.	%			
Burkina Faso									
Total No. of positive malaria cases^a^	994	…	388	…	606	…	1.56^b^	1.13–2.15	.006
Total No. of AL treatment given (% out of cases)	761	76.6	302	77.8	459	75.7	1.08^b^	.94–1.25	.274
Total No. of women tested positive at least once	568	31.6	237	26.3	331	36.8	1.67^c^	1.22–2.30	.002
The Gambia									
Total No. of positive malaria cases^a^	87	…	15	…	72	…	4.55^b^	2.48–8.34	<.001
Total No. of AL treatment given (% out of cases)	71	81.6	12	80.0	59	81.9	1.23^b^	.66–2.29	.515
Total No. of women tested positive at least once	75	3.8	15	1.6	60	6.0	3.94^c^	2.05–7.59	<.001
Benin									
Total No. of positive malaria cases^a^	224	…	33	…	191	…	4.67^b^	2.93–7.44	<.001
Total No. of AL treatment given (% out of cases)	186	83.0	14	42.4	172	90.1	1.71^b^	.99–2.94	.055
Total No. of women tested positive at least once	164	16.9	20	4.7	144	26.6	7.53^c^	4.49–12.64	<.001

Abbreviations: AL, artemether-lumefantrine; CI, confidence interval; OR, odds ratio; RR, rate ratio.

^a^Woman can be positive more than once during pregnancy.

^b^RRs obtained from Poisson regression models.

^c^ORs obtained from logistic regression models.

### The Effect of the Intervention on Placenta Malaria

Data from placental biopsies were available for 88% (3171/3585) of deliveries in Burkina Faso and The Gambia. There was no difference between intervention and control arms in terms of placenta malaria or for different categories of infection (Table 3), and this did not change after adjusting for season of birth, parity, and number of IPTp-SP administered (adjusted OR [aOR], 1.06 [95% CI, .78–1.44]; *P* = .72) (Table 4). Similar results were observed at country level (Supplementary Tables 2 and 3).

**Table 3.** Placental Malaria in Burkina Faso and The Gambia

Histology	Control		Intervention		Odds Ratio	95% CI	*P* Value
	No.	%	No.	%			
No. with biopsy data	1544		1622		…	…	…
Placental histology							
Any infection (acute, chronic, or past)	494	32	533	33	1.09	.80–1.48	.588
No infection	1050	68	1089	67	…	…	…
Active infection (acute or chronic)	64	4	65	4	0.99	.66–1.49	.974
No active infection	1480	96	1557	96	…	…	…
Past or chronic infection	472	31	509	31	1.09	.81–1.46	.571
No past or chronic infection	1072	69	1113	69	…	…	…

Abbreviation: CI, confidence interval.

**Table 4.** Overall Adjusted Analysis for Placental Malaria in The Gambia and Burkina Faso

Characteristic	No.	Positive, No. (%)	Unadjusted OR	95% CI	*P* Value	Adjusted OR^a^	95% CI	*P* Value
Arm								
Intervention	1621	533 (33)	…	…	…	…	…	…
Control	1543	494 (32)	1.09	.80–1.48	.588	1.06	.78–1.44	.722
Seasonality								
Delivery in rainy reason	1684	440 (26)	0.71	.59–.84	<.001	0.59	.49–.71	<.001
Delivery in dry season	1480	587 (40)	1	…	…	…	…	…
Gravida								
First or second pregnancy	1124	451 (40)	…	…	…	…	…	…
>2 pregnancies	2040	576 (28)	0.47	.39–.56	<.001	0.47	.39–.57	<.001
No. of SP doses								
0	9	1 (11)	0.90	.10–.77	…	0.54	.06–5.14	…
1	496	140 (28)	1	…	…	1	…	…
2	1892	571 (30)	0.87	.66–1.14	…	0.85	.65–1.12	…
3	460	203 (44)	0.41	.29–.57	…	0.37	.26–.53	…
4	251	95 (38)	0.23	.16–.34	…	0.23	.15–.34	…
5	50	15 (30)	0.15	.08–.30	…	0.13	.07–.27	…
6	6	2 (33)	0.15	.03–.90	<.001	0.10	.01–.64	<.001
No. of AL treatments given								
0	2651	695 (26)	1	…	…	1	…	…
1	364	223 (61)	2.10	1.62–2.72	…	2.01	1.53–2.64	…
2	75	75 (70)	2.60	1.66–4.08	…	1.86	1.15–3.02	…
≥3	34	34 (81)	4.52	2.01–10.15	<.001	2.98	1.28–6.95	<.001

Data are shown for any infection (acute, chronic, or past).

Abbreviations: AL, artemether-lumefantrine; CI, confidence interval; OR, odds ratio; SP, sulfadoxine-pyrimethamine.

^a^Adjusted for all other variables in the table.

### The Effect of the Intervention on Other Secondary Outcomes at Delivery

There was no difference between arms in peripheral maternal infection (OR, 0.97 [95% CI, .78–1.21]; *P* = .7), even after adjusting for several factors (aOR, 0.92 [95% CI, .74–1.15]; *P* = .4) (Table 5; country-specific data in Supplementary Table 3). Anemia (OR, 1.09 [95% CI, .92–1.28]; *P* = .3; Table 6 and Supplementary Table 4), mean hemoglobin (mean difference, 0.01 [95% CI, –.29 to .31]; *P* = .9), low birth weight (OR, 1.06 [95% CI, .82–1.38]; *P* = .6; Table 7 and Supplementary Table 5), and adverse pregnancy outcomes (Table 8 and Supplementary Table 6) did not differ between study arms. Nevertheless, in Burkina Faso, the odds of miscarriage tended to be higher in the intervention arm (OR, 4.54 [95% CI, .98–21.05]; *P* = .054; Supplementary Table 6*A*). Among these women, 3 had a malaria infection at least once during pregnancy (2 in the intervention arm and 1 in the control arm), and 2 received 1 course of AL (1 in each arm), with no drug-related serious adverse event recorded. There was no evidence that taking AL during pregnancy was associated with any adverse pregnancy outcome (at least 1 course of AL: OR, 0.82 [95% CI, .35–1.93]; *P* = .656).

**Table 5.** Overall Adjusted Analysis for Maternal Peripheral Infection at Delivery (as Measured by Polymerase Chain Reaction)

Characteristic	No.	Positive, No. (%)	Unadjusted OR	95% CI	*P* Value	Adjusted OR^a^	95% CI	*P* Value
Arm								
Intervention	1951	208 (10.66)	1	…	…	1	…	…
Control	1826	200 (10.95)	0.97	.78–1.21	.798	0.92	.74–1.15	.456
Seasonality								
Delivery in rainy reason	1796	125 (6.96)	2.94	2.34–3.69	<.001	2.84	2.25–3.58	<.001
Delivery in dry season	1981	283 (14.29)	1	…	…	1	…	…
Gravida								
First or second pregnancy	1329	165 (12.42)	1	…	…	1	…	…
>2 pregnancies	2448	243 (9.93)	0.76	.61–.84	.012	0.83	.66–1.04	.101
No. of AL treatments given								
0	3107	273 (8.79)	1	…	…	1	…	…
1	478	88 (18.41)	1.43	1.09–1.88	…	1.33	1.00–1.77	…
2	145	33 (22.76)	1.76	1.16–2.66	…	1.76	1.13–2.73	…
≥3 (max 6)	47	14 (29.79)	2.35	1.23–4.48	<.001	2.26	1.15–4.47	.006
No. of SP doses								
0	4	0 (0)	…	…	…	…	…	…
1	677	83 (12.26)	1	…	…	1	…	…
2	2192	219 (9.99)	0.72	.54–.96	…	0.74	.55–.98	…
3	531	50 (9.42)	0.30	.20–.44	…	0.37	.24–.56	…
4	298	46 (15.44)	0.47	.31–.71	…	0.55	.36–.85	…
5	67	9 (13.43)	0.39	.18–.84	…	0.44	.20–.94	…
6	8	1 (12.50)	0.38	.05–3.15	<.001	0.28	.03–2.42	<.001

Abbreviations: AL, artemether-lumefantrine; CI, confidence interval; OR, odds ratio; SP, sulfadoxine-pyrimethamine.

^a^Adjusted for all other variables in the table.

**Table 6.** Anemia at Delivery, by Study Arm (All Countries)

Characteristic	No.	Anemic^a^, No. (%)	OR	95% CI	*P* Value	Adjusted	95% CI	*P* Value
Arm								
Intervention	1989	861 (43)	1	…	…	1	…	…
Control	2167	979 (45)	1.08	.92–1.28	.351	1.09	.92–1.28	.321
Seasonality								
Delivery in rainy season	2194	1089 (50)	1	…	…	1	…	…
Delivery in dry season	1962	751 (38)	0.72	.63–.82	<.001	0.73	.64–.83	<.001
Gravida								
First or second pregnancy	1480	634 (43)	1	…	…	1	…	…
>2 pregnancies	2674	1205 (45)	1.13	.99–1.30	.078	1.14	1.00–1.32	.052
No. of AL treatments given								
0	3458	1623 (47)	1	…	…	1	…	…
1	505	147 (29)	0.85	.68–1.06	…	0.83	.66–1.04	…
2	146	56 (38)	1.54	1.07–2.21	…	1.58	1.09–2.28	…
≥3 (max 6)	47	14 (30)	1.23	.64–2.35	…	1.26	.65–2.41	…
					.029			.017
No. of SP doses								
1	754	407 (54)	1	…	…	1	…	…
2	2454	1187 (48)	0.80	.67–.95	…	0.81	.68–.96	…
3	549	147 (27)	0.68	.52–.89	…	0.72	.54–.95	…
4	312	74 (24)	0.70	.50–.98	…	0.73	.52–1.02	…
5	69	20 (29)	0.94	.53–1.66	…	0.99	.99–1.75	…
6	8	2 (25)	0.76	.15–3.86	…	0.78	.15–4.06	…
					.022			.065

Abbreviations: AL, artemether-lumefantrine; CI, confidence interval; OR, odds ratio; SP, sulfadoxine-pyrimethamine.

^a^Anemia defined as hemoglobin level <11 g/dL.

**Table 7.** Low Birth Weight, by Study Arm (All Countries)

Characteristic	No.	LBW, No. (%)	OR	95% CI	*P* Value	Adjusted	95% CI	*P* Value
Arm								
Intervention	2127	219 (10)	1	…	…	1	…	…
Control	1950	201 (10)	1.06	.82–1.38	.643	1.06	.81–1.38	.695
Seasonality								
Delivery in rainy season	2159	213 (10)	1	…	…	1	…	…
Delivery in dry season	1918	207 (11)	1.09	.89–1.34	.417	1.13	.92–1.40	.247
Gravida								
First or second pregnancy	1456	212 (15)	1	…	…	1	…	…
>2 pregnancies	2619	208 (8)	0.50	.40–.61	<.001	0.50	.40–.61	<.001
No. of AL treatments given								
0	3393	334 (10)	1	…	…	1	…	…
1	493	62 (13)	1.36	1.00–1.85	…	1.24	.91–1.71	…
2	144	16 (11)	1.18	.68–2.04	…	0.88	.50–1.55	…
≥3 (max 6)	47	8 (17)	1.87	.84–4.12	…	1.34	.62–3.12	…
					.128			.429
No. of SP doses								
1	731	111 (15)	1	…	…	1	…	…
2	2415	230 (10)	0.55	.28–6.96	…	0.54	.42–.70	…
3	539	49 (9)	0.45	.43–.71	…	0.44	.30–.66	…
4	305	21 (7)	0.32	.30–.66	…	0.33	.19–.55	…
5	69	7 (10)	0.46	.19–.54	…	0.43	.18–.55	…
6	0	0 (0)	…	…	…	…	…	…
					<.001			<.001

Abbreviations: AL, artemether-lumefantrine; CI, confidence interval; LBW, low birth weight; OR, odds ratio; SP, sulfadoxine-pyrimethamine.

**Table 8.** Adverse Outcome of Pregnancy, by Study Arm (All Countries)

Outcome	Control		Intervention		OR/Mean Difference	95% CI	*P* Value
	No.	%	No.	%			
Anemia							
Hb <11 g/dL	861	43	979	45	1.09^a^	.92–1.28	.351
Hb ≥11 g/dL	1128	57	1188	55	…	…	…
Mean Hb (SD)	11.15	0.71	11.14	0.73	0.01^b^	–.29 to .31	.944
Low birth weight (<2500 g)							
<2500	201	10	219	10	1.06^a^	.82–1.38	.643
≥2500	1749	90	1908	90	…	…	…
Mean birth weight, kg (SD)	2.98	0.17	2.94	0.14	0.04^b^	–.04 to .10	.254
Adverse pregnancy outcomes							
Congenital abnormalities	16	0.8	29	1.3	1.70^a^	.92–3.15	.089
Miscarriage	16	0.7	16	0.7	0.98^a^	.45–2.15	.959
Preterm birth	60	3	70	3	1.06^a^	.68–1.67	.793
Stillbirth	39	2	47	2	1.05^a^	.64–1.72	.855
Miscarriage, preterm, or stillbirth	99	4	116	5	1.10^a^	.79–1.52	.582
Deaths							
Perinatal death	21	1	24	1	1.03^a^	.57–1.85)	.933
Maternal death	6	0.3	3	0.1	0.46^a^	.12–1.86)	.279
Perinatal death, miscarriage, preterm, or stillbirth	114	5	131	5	1.07^a^	.79–1.45)	.654

Abbreviations: CI, confidence interval; Hb, hemoglobin; OR, odds ratio; SD, standard deviation.

aOR.

bMean difference.

There were 9 maternal deaths (6 in the control arm and 3 in the intervention arm) and 45 perinatal deaths that occurred at time of delivery (21 in control vs 24 in intervention arm). Causes attributed to the SAEs, including maternal and perinatal death, are reported in Supplementary Table 8.

### ANC Attendance and IPTp-SP Uptake During Pregnancy

Antenatal clinic attendance (scheduled visits) was significantly higher in the intervention arm in Burkina Faso but not in The Gambia and Benin (Table 9 and Supplementary Table 7). However, IPTp-SP coverage (ie, mean number of doses and percentage of women who received at least 2 or 4 doses) was not significantly different between intervention and control arms.

**Table 9.** Summary Estimates of the Effect of the Intervention on Antenatal Clinic Attendance and Intermittent Preventive Treatment With Sulfadoxine-Pyrimethamine Coverage, by Country^a^

Intervention	Burkina Faso			The Gambia			Benin		
	OR/IRR	95% CI	*P* Value	OR/IRR	95% CI	*P* Value	OR/IRR	95% CI	*P* Value
ANC visits									
At least 2 scheduled visits	…	…	…	1.03^b^	.66–1.62	.881	0.88^b^	.63–1.24	.471
At least 4 scheduled visits	1.62^b^	1.02–2.59	.041	…	…	…	…	…	…
Mean No. of scheduled visits (SD)	1.08^c^	1.00–1.17	.045	1.00^c^	.93–1.09	.912	0.98^c^	.93–1.04	.484
Mean No. of unscheduled visits (SD)	0.90^c^	.63–1.30	.589	1.34^c^	.80–2.22	.266	1.07^c^	.61–1.89	.81
Mean No. of any ANC visits (SD)	1.06^c^	.96–1.16	.258	1.07c	.90–1.27	.445	0.99^c^	.91–1.07	.781
IPTp-SP coverage									
Mean No. of SP doses	1.04^c^	.97–1.10	.294	1.01^c^	.97–1.05	.611	0.98^c^	.92–1.03	.387
At least 2 doses of SP	1.29^b^	.96–1.73	.093	1.01^b^	.70–1.46	.938	0.87^b^	.62–1.22	.418
At least 4 doses of SP	1.14^b^	.76–1.72	.517	…	…	…	…	…	…

Abbreviations: ANC, antenatal clinic; CI, confidence interval; IPTp-SP, intermittent preventive treatment in pregnancy with sulfadoxine-pyrimethamine; IRR, incidence rate ratio; OR, odds ratio; SD, standard deviation; SP, sulfadoxine-pyrimethamine.

^a^IPTp-SP policies vary between countries; thus, data are not pooled.

bOR.

cIRR.

### The Effect of IPTp-SP on Pregnancy Outcomes

Increasing number of IPTp-SP doses was associated with a significantly lower risk of placenta malaria (Table 4), mainly because of Burkina Faso where 49.2% (886/1800) of women had received ≥3 IPTp-SP doses (Supplementary Table 3*A*), whereas in The Gambia this figure was only 3.7% (73/1960) (Supplementary Table 3*B*). Placenta malaria occurred significantly less during the rainy than the dry season (aOR, 0.59 [95% CI, .49–.71]; *P* < .001), and was more frequent with increasing number of AL treatments (Table 4).

Increasing number of IPTp-SP doses tended to decrease the risk of anemia at delivery (*P* = .06), whereas increasing number of AL treatments had the opposite effect (*P* = .02) (Table 6 and Supplementary Table 4). Similarly, the risk of low birth weight decreased significantly with the increasing number of IPTp-SP doses (*P* < .001; Table 7 and Supplementary Table 5).

## DISCUSSION

Adding CSST by CHWs to the standard IPTp-SP at ANC did not reduce the risk of placental malaria or peripheral malaria infection at delivery. The intervention also aimed at increasing ANC attendance, particularly in early pregnancy, and at identifying and treating infections between scheduled ANC visits. Scheduled ANC attendance did improve in Burkina Faso, suggesting that the intervention had the expected effect. During the trial, the 2013 WHO recommendations of at least 4 ANC visits and of administering IPTp-SP at each of them [17] had been implemented in Burkina Faso, with some women having as many as 6–7 scheduled ANC visits. In The Gambia and Benin, the national policy was still 2 scheduled visits; thus, we did not expect an increase in the number of ANC visits but rather an increase in coverage of 2 ANC visits. ANC attendance remained extremely low in Benin, where only slightly more than half of the women had 2 visits, and this may reflect the general poor attendance in southern Benin. In The Gambia, ANC attendance was already high, with 80% of the women attending at least 2 scheduled visits, and this may explain why the intervention did not have the expected effect.

In all countries, the higher number of malaria infections diagnosed in the intervention group and the high proportion of malaria-positive women treated with AL indicate that CHWs implemented the intervention according to the instructions received. In Burkina Faso, RDT sensitivity and specificity performed by CHWs and compared to microscopy was 81.5% (95% CI, 67.9%–90.2%) and 92.1% (95% CI, 89.9%–93.9%), respectively [14], further confirming CHWs can both correctly use RDTs and adhere to test results and treatment guidelines [18]. Previous studies have shown that CHWs are able to diagnose and treat malaria in children [19–22] and are able to distribute IPTp to pregnant women [23].

Though the intervention did not have a direct effect on the prevalence of past placenta malaria, probably because of infections before the first ANC visit, one would have expected a reduced risk of active or chronic placenta infections. This was not the case, and this may have been due to low adherence to AL treatment. However, CHWs who visited treated women 3 days after CSST, and 96% of women reported having completed the full course; in contrast, a qualitative study carried out in the same study area reported low adherence to antimalarial treatment by pregnant women despite good knowledge about malaria in pregnancy [24].

Increasing doses of IPTp-SP significantly decreased the risk of placental malaria. This effect was mainly seen in Burkina Faso, where women received up to 6 IPTp-SP doses. In The Gambia and Benin, the large majority of women had taken only 2 IPTp-SP doses and in both countries malaria prevalence was lower than in Burkina Faso. In these 2 countries, CSST should have had some effect as the time between IPTp-SP doses was longer than in Burkina Faso, so diagnosing and treating infections during this period should have been beneficial. Malaria screening by the CHWs was done with an RDT whose detection threshold is at most 200 parasites/μL [25]. However, the large majority of infections during pregnancy are asymptomatic, with low parasite densities, often not detected by microscopy or RDT [26]. Therefore, measurements of the efficacy of intermittent screening and treatment may be limited by the sensitivity of current RDTs [10]. The decreased risk of low birth weight with increasing IPTp-SP doses and the borderline decrease of maternal anemia at delivery confirms the meta-analysis of 7 trials carried out in sub-Saharan Africa that reported a lower risk of low birth weight, maternal anemia, and placental malaria in women who received ≥3 IPTp-SP doses [27]. These results were used to support the WHOs’ recommendation of administering IPTp-SP at each ANC, provided the doses are at least a month apart. The current trial data support this recommendation.

Pregnant women in the intervention arm had a higher risk of testing malaria positive, not because they had a higher risk of being infected, but rather because they were tested more frequently. Considering that most infections diagnosed in the intervention arm were treated with AL and that the risk of placenta; malaria increased with the number of AL treatments administered, these infections probably represented a small proportion of all infections acquired during pregnancy and it was the undiagnosed infections that had a significant effect on the occurrence of placenta malaria. Receiving multiple AL treatments is probably a marker of a higher malaria risk. There is some controversy on the importance of low-density malaria infections during pregnancy, associated with anemia, lower mean hemoglobin, low birth weight, and premature births in some studies [28] but not in others [26]. Our results indicate that such infections are important and that, until better diagnostic tests than standard RDTs become available, systematic treatment as many times as possible of all pregnant women until delivery is the best approach.

A major strength of this study was the selection of countries based on their varying malaria endemicity: low (The Gambia) vs high (Burkina Faso and Benin), and with varying degrees of SP resistance (high in Benin and moderate in The Gambia and Burkina Faso), which us enables to generalize study findings to West Africa and possibly other sub-Saharan African countries. The study was powered for individual countries for the overall primary outcome; thus, we were able to at least fully investigate the effect of the intervention on placental malaria for the study sites in The Gambia and Burkina Faso. Unfortunately, this was not possible for Benin.

The significant number of adverse pregnancy outcomes in both study arms highlights the poor access to timely and adequate care for women in rural and remote areas in sub-Saharan Africa. Both maternal anemia and low birth weight (45% and 10%, respectively) were below the West African regional estimates, 56% (95% CI, 46%–62%) [29] and 14% (https://data.unicef.org/topic/nutrition/low-birthweight/). Being in a trial may have influenced women’s healthcare-seeking behavior, which may explain these lower estimates. There were 45 perinatal deaths and 9 maternal deaths with no difference in mortality between the study arms. Pooling the data together gives a perinatal mortality rate of 10.77 per 1000 live births, and a maternal mortality rate of 215 per 100000 live births. These remain high levels, but comparisons with other sources [30–32] should be interpreted with caution as they were estimated at time of delivery while the neonatal and maternal mortality usually include days 28 and 42 postpartum, respectively. It is reassuring that no adverse events were associated with AL treatment, providing further safety data for AL treatment in the second and third trimester of pregnancy.

IST has been extensively evaluated as an alternative to IPTp-SP, with mixed results [8–10]. We decided to combine these 2 interventions, with the aim of providing additional protection against malaria between ANC visits. Despite the significantly higher number of women treated for malaria in the intervention arm, none of the trial outcomes differed between study arms. Treatment with a long-acting artemisinin-based combination therapy such as dihydroartemisinin-piperaquine instead of AL would possibly have had a better outcome. Indeed, the number of AL treatments administered was strongly associated with placental malaria, peripheral infection, and anemia, indicating that treated women had a higher risk of being reinfected over a relatively short period. Such a risk could have been lowered by a treatment with a much longer posttreatment prophylactic period. That the length of the prophylaxis period is more important than treating diagnosed infections is shown by the beneficial effect of increasing IPTp-SP doses on different pregnancy outcomes, including low birth weight. This may change when more sensitive diagnostic tests become available but, for now, increasing the number of IPTp-SP doses given during pregnancy is a priority.

## Supplementary Data

Supplementary materials are available at *Clinical Infectious Diseases* online. Consisting of data provided by the authors to benefit the reader, the posted materials are not copyedited and are the sole responsibility of the authors, so questions or comments should be addressed to the corresponding author.

Supplementary Information

## Notes

***Acknowledgments*.** We thank all the study participants of the COSMIC trial, in particular the pregnant women and the CHWs who carried out the intervention. We also thank the research and field staff of The Medical Research Council Unit, The Gambia, Unité de Recherche Clinique de Nanoro, Burkina Faso and Centre de Recherches Entomologiques de Cotonou, Benin and the Ministries of Health in the respective countries who supported the study. Staff of the Department of Pathology at the Edward Francis Small Teaching Hospital, The Gambia whose support we acknowledge, prepared the placental histology slides.

***Financial support*.** This work was supported by the European Community’s Seventh Framework Programme (grant agreement number 305662). Novartis kindly provided the study drug, Coartem.

***Potential conflicts of interest.*** All authors: No reported conflicts. All authors have submitted the ICMJE Form for Disclosure of Potential Conflicts of Interest. Conflicts that the editors consider relevant to the content of the manuscript have been disclosed.

## Notes

***COSMIC Consortium.*** Medical Research Council, The Gambia/London School of Hygiene and Tropical Medicine: Susana Scott, Umberto D’Alessandro, Lindsay Kendall, John Bradley, Kalifa Bojang, Simon Correa, Fanta Njie. Institut de Recherche en Sciences de la Santé–Unité de Recherche Clinique de Nanoro, Burkina Faso: Halidou Tinto, Maminata Traore-Coulibaly, Hamtandi Magloire Natama, Ousmane Traoré, Innocent Valea. Centre de Recherches Entomologiques de Cotonou, Benin: Alain Nahum, Daniel Ahounou, Francis Bohissou, Gethaime Sondjo, Carine Agbowai. Academic Medical Centre, The Netherlands: Petra Mens, Esmée Ruizendaal, Henk Schallig. Institute of Tropical Medicine, Belgium: Susan Dierickx, Koen Peeters Grietens. Imperial College London, UK: Laetitia Duval, Lesong Conteh. Institut de Recherche en Sciences de la Santé, Burkina Faso: Maxime Drabo. Special Programme for Research and Training in Tropical Diseases, WHO, Switzerland: Jamie Guth, Franco Pagnoni.
