# Supplementary material for: Community-based Malaria Screening and Treatment for Pregnant Women Receiving Standard Intermittent Preventive Treatment With Sulfadoxine-Pyrimethamine: A Multicenter (The Gambia, Burkina Faso, and Benin) Cluster-randomized Controlled Trial
Source: Clin Infect Dis. 2018 Jun 29;68(4):586–96. doi: 10.1093/cid/ciy522 (PMC6355825; doi:10.1093/cid/ciy522)
Supplement: Supplementary Information [file ciy522_suppl_supplementary_information.docx]

**Supplementary Information**

**Figure S1: Flow chart of pregnant woman cohort by country**

S1a: Burkina Faso

S1b: The Gambia

S1c: Benin

**Table S1a: Home visits in the intervention arm only**

|  | **Burkina Faso** | | **Gambia** |  | **Benin** |  |
| --- | --- | --- | --- | --- | --- | --- |
|  | **N** | **%** | **N** | **%** | **N** | **%** |
| **Home visits (intervention only)** |  |  |  |  |  |  |
| Total no. of women who had a home visits | 860 | 96% | 978 | 97% | 474 | 87.45% |
| Reasons no home visit |  |  |  |  |  |  |
| withdrew | 11 | 27.50% | 2 | 6.67% | 27 | 39.71% |
| moved | 2 | 5.00% | 5 | 16.67% | 11 | 16.18% |
| Wrongly recruited | 0 | 0.00% | 3 | 10.00% | 2 | 2.94% |
| Women died before home visits | 0 | 0.00% | 1 | 3.33% | 0 | 0.00% |
| NK | 27 | 67.50% | 19 | 63.33% | 28 | 41.18% |
| Total no of home visits | 2511 |  | 3432 |  | 1293 |  |
| Median no per women (IQR) | 3 | (2-4) | 4 | (3-4) | 3 | (2-4) |
| **Total no of malaria cases diagnosed at home visits** | 307 |  | 45 |  | 165 |  |
| Tested positive once | 187 | 77.27% | 43 | 97.73% | 109 | 80.15% |
| 2 times | 46 | 19.01% | 1 | 2.27% | 25 | 18.38% |
| 3 times | 8 | 3.31% | 0 | 0.00% | 2 | 1.47% |
| 4 times | 1 | 0.41% | 0 | 0.00% | 0 | 0.00% |
| Total women who tested positive at least once (% out of total in recruited) | 242 | 26.89% | 44 | 4.37% | 136 | 25.09% |
| **Total no of AL treatments given at home visit (% out of total positive)** | 238 | 77.52% | 37 | 82.22% | 163 | 98.79% |
| AL given once | 172 | 84.73% | 37 | 100.00% | 107 | 79.85% |
| 2 times | 27 | 13.30% | 0 | 0.00% | 25 | 18.66% |
| 3 times | 4 | 1.97% | 0 | 0.00% | 2 | 1.49% |
| Total no of AL given at least once | 203 | 83.88% | 37 | 84.09% | 134 | 98.53% |

**Table S1b: Malaria diagnosed at Clinic visits – both arms**

| **Burkina Faso** | **Overall** |  | **Control** |  | **Intervention** | | **OR/RR** | **95% CI** | **p-value** |
| --- | --- | --- | --- | --- | --- | --- | --- | --- | --- |
|  | **N** | **%** | **N** | **%** | **N** | **%** |  |  |  |
| Total Malaria cases diagnosed at ANC | 687 |  | 388 |  | 299 |  |  |  |  |
| 1 time | 283 | 66.12% | 148 | 62.45% | 135 | 70.68% |  |  |  |
| 2 time | 84 | 19.63% | 53 | 22.36% | 31 | 16.23% |  |  |  |
| 3 times | 37 | 8.64% | 23 | 9.70% | 14 | 7.33% |  |  |  |
| 4 times | 10 | 2.34% | 6 | 2.53% | 4 | 2.09% |  |  |  |
| 5 times | 6 | 1.40% | 3 | 1.27% | 3 | 1.57% |  |  |  |
| 6 times | 5 | 1.17% | 3 | 1.27% | 2 | 1.05% |  |  |  |
| 8 times | 2 | 0.47% | 1 | 0.42% | 1 | 0.52% |  |  |  |
| 9 times | 1 | 0.23% | 0 | 0.00% | 1 | 0.52% | 0.77 | (0.51,1.16) | 0.214 |
| No women diagnosed at least once at ANC (% out of recruited) | 428 | 23.78% | 237 | 26.33% | 191 | 21.22% | 0.76 | (0.51,1.12) | 0.167 |

| **The Gambia** | **Overall** |  | **Control** |  | **Intervention** | | **OR/RR** | **95% CI** | **p-value** |
| --- | --- | --- | --- | --- | --- | --- | --- | --- | --- |
|  | **N** | **%** | **N** | **%** | **N** | **%** |  |  |  |
| Total Malaria diagnosed at ANC | 42 |  | 15 |  | 29 |  |  |  |  |
| 1 time | 39 | 97.50% | 15 | 100.00% | 24 | 96.00% |  |  |  |
| twice | 0 | 0.00% | 0 | 0.00% | 0 | 0.00% |  |  |  |
| 3 times | 1 | 2.50% | 0 | 0.00% | 1 | 4.00% |  |  |  |
| No women diagnosed at least once at ANC (% out of recruited) | 40 | 2.04% | 15 | 1.58% | 25 | 2.48% | 1.59 | (0.83,3.03) | 0.16 |

| **Benin** | **Overall** |  | **Control** |  | **Intervention** | | **OR/RR** | **95% CI** | **p-value** |
| --- | --- | --- | --- | --- | --- | --- | --- | --- | --- |
|  | **N** | **%** | **N** | **%** | **N** | **%** |  |  |  |
| Total Malaria diagnosed at ANC | 59 |  | 33 |  | 26 |  |  |  |  |
| 1 time | 11 | 32.35% | 7 | 35.00% | 4 | 28.57% |  |  |  |
| twice | 21 | 61.76% | 13 | 65.00% | 8 | 57.14% |  |  |  |
| 3 times | 2 | 5.88% | 0 | 0.00% | 2 | 14.29% | 0.65 | (0.26,1.66) | 0.368 |
| No women diagnosed at least once at ANC (% out of recruited) | 34 | 3.50% | 20 | 4.66% | 14 | 2.58% | 0.55 | (0.23,1.29) | 0.168 |

**Table S2: Placental malaria and peripheral infection at delivery by country**

**S2a: Burkina Faso**

|  | **Control** |  | **Intervention** | | **Odds ratio / IRR** | **(95% CI)** | **P-value** |
| --- | --- | --- | --- | --- | --- | --- | --- |
|  | N | % | N | % |  |  |  |
| N with Biopsy data | 698 |  | 716 |  |  |  |  |
| **Placental Histology** |  |  |  |  |  |  |  |
| Any infection (acute, chronic or past) | 408 | (58.45) | 401 | (56.01) | 0.91 | (0.64,1.30) | 0.593 |
| No infection | 290 | (41.55) | 315 | (43.99) | 1 |  |  |
| Active Infection (acute or chronic) | 52 | (7.45) | 46 | (6.42) | 0.86 | (0.53,1.39) | 0.526 |
| No active infection | 646 | (92.55) | 670 | (93.58) | 1 |  |  |
| Past or chronic infection | 395 | (56.59) | 392 | (54.75) | 0.93 | (0.66,1.31) | 0.685 |
| No past or chronic infection | 303 | (43.41) | 324 | (45.25) | 1 |  |  |
| **Maternal Peripheral infection (PCR)** |  |  |  |  |  |  |  |
| Positive | 145 | (17.53) | 148 | (17.83) | 1.02 | (0.79,1.32) | 0.875 |
| Negative | 682 | (82.47) | 682 | (82.17) | 1 |  |  |
| **Malaria infection during pregnancy (microscopy)** | |  |  |  |  |  |  |
| Mean number of times blood slides taken (SD) | 4.78 | (0.57) | 7.73 | (0.74) | 1.62 | (1.50,1.74) | <0.001 |
| **Number of positive slides per women** |  |  |  |  |  |  |  |
| 0 | 453 | (50.45) | 432 | (48) |  |  |  |
| 1 | 297 | (33.07) | 298 | (33.11) |  |  |  |
| 2 | 95 | (10.58) | 97 | (10.78) |  |  |  |
| 3 | 40 | (4.45) | 48 | (5.33) |  |  |  |
| 4 | 7 | (0.78) | 18 | (2) |  |  |  |
| 5 | 4 | (0.45) | 5 | (0.56) |  |  |  |
| 6 | 2 | (0.22) | 2 | (0.22) |  |  |  |
| Total number of slides positive | 667 | (49.55) | 745 | (52) | 1.11 | (0.97,1.28) | 0.133 |
| At least 1 infection during pregnancy | 445 | (49.44) | 468 | (52) | 1.11 | (0.92,1.34) | 0.291 |

**S2b: The Gambia**

|  | **Control** |  | **Intervention** | | **Odds ratio / IRR** | **(95% CI)** | **P-value** |
| --- | --- | --- | --- | --- | --- | --- | --- |
|  | N | % | N | % |  |  |  |
| N with Biopsy data | 850 |  | 907 |  |  |  |  |
| **Placental Histology** |  |  |  |  |  |  |  |
| Any infection (acute, chronic or past) | 89 | (10.47) | 133 | (14.66) | 1.36 | (0.81,2.28) | 0.24 |
| No infection | 761 | (89.53) | 774 | (85.34) | 1 |  |  |
| Active Infection (acute or chronic) | 12 | (1.41) | 19 | (2.09) | 1.49 | (0.72,3.10) | 0.28 |
| No active infection | 838 | (98.59) | 888 | (97.91) | 1 |  |  |
| Past or chronic infection | 80 | (9.41) | 118 | (13.01) | 1.33 | (0.81,2.19) | 0.254 |
| No past or chronic infection | 770 | (90.59) | 789 | (86.99) | 1 |  |  |
| **Maternal Peripheral infection (PCR)** |  |  |  |  |  |  |  |
| Positive | 19 | (48.72) | 20 | (51.28) | 1.05 | (0.52,2.10) | 0.902 |
| Negative | 718 | (49.08) | 745 | (50.92) | 1 |  |  |
| **Malaria infection during pregnancy (microscopy)** | |  |  |  |  |  |  |
| Mean number of times blood slides taken (SD) | 3.15 | (0.33) | 6.53 | (0.39) | 2.08 | (1.98,2.19) | <0.001 |
| **Number of positive slides per women** |  |  |  |  |  |  |  |
| 0 | 861 | (90.82) | 881 | (87.49) |  |  |  |
| 1 | 29 | (3.06) | 43 | (4.27) |  |  |  |
| 2 | 54 | (5.7) | 69 | (6.85) |  |  |  |
| 3 | 3 | (0.32) | 7 | (0.7) |  |  |  |
| 4 | 1 | (0.11) | 6 | (0.6) |  |  |  |
| 6 | 0 | (0) | 1 | (0.1) |  |  |  |
| Total number of slides positive | 150 | (9.18) | 232 | (12.51) | 1.46 | (0.99,2.13) | 0.053 |
| At least 1 infection during pregnancy | 87 | (9.14) | 126 | (12.5) | 1.44 | (0.99,2.10) | 0.053 |

**S2c: Benin**

|  | **Control** |  | **Intervention** | | **Odds ratio / IRR** | **(95% CI)** | **p-value** |
| --- | --- | --- | --- | --- | --- | --- | --- |
|  | N | % | N | % |  |  |  |
| **Maternal Peripheral infection (PCR) (% of total enrolled)** |  |  |  |  |  |  |  |
| Positive | 36 | (13.74) | 40 | (11.2) | 0.79 | (0.49,1.28) | 0.34 |
| Negative | 226 | (86.26) | 317 | (88.8) | 1 |  |  |
| **Malaria infection during pregnancy (microscopy)** | |  |  |  |  |  |  |
| Mean number of times blood slides taken (SD) | 2.25 | (0.39) | 4.75 | (0.7) | 2.04 | (1.87,2.23) | <0.001 |
| **Number of positive slides per women** |  |  |  |  |  |  |  |
| 0 | 269 | (62.85) | 315 | (58.12) |  |  |  |
| 1 | 121 | (28.27) | 160 | (29.52) |  |  |  |
| 2 | 33 | (7.71) | 52 | (9.59) |  |  |  |
| 3 | 5 | (1.17) | 11 | (2.03) |  |  |  |
| 4 | 0 | (0) | 4 | (4) |  |  |  |
| Total number of slide positives | 202 | (37.15) | 313 | (41.88) | 1.22 | (0.98,1.53) | 0.079 |
| At least 1 infection during pregnancy | 159 | (37.15) | 227 | (41.88) | 1.23 | (0.93,1.63) | 0.152 |

**Table S3: Adjusted analysis for placental malaria (any type) and maternal peripheral infection at time of delivery**

**S3a: Burkina Faso**

1. Any malaria infection (active, chronic or both)

| **Any infection (acute, chronic or past)** | N | **% positive** | **Unadjusted Odds ratio** | **(95% CI)** | **P-value** | **Adjusted** | **(95% CI)** | **p-value** |
| --- | --- | --- | --- | --- | --- | --- | --- | --- |
| **Arm** |  |  |  |  |  |  |  |  |
| Intervention | 716 | (56.01) | 0.91 | (0.64,1.30) | 0.593 | 0.91 | (0.64,1.30) | 0.615 |
| Control | 698 | (58.45) | 1 |  |  | 1 |  |  |
| **Seasonality** |  |  |  |  |  |  |  |  |
| Born in rain reason (July-December) | 615 | (51.22) | 0.67 | (0.54,0.84) | <0.001 | 0.53 | (0.42,0.67) | <0.001 |
| Born in non-rain season (Jan-June) | 795 | (61.64) | 1 |  |  | 1 |  |  |
| **Gravida** |  |  |  |  |  |  |  |  |
| First or second pregnancy | 488 | (68.65) | 2.09 | (1.65,2.65) | <0.001 | 2.08 | (1.61,2.68) | <0.001 |
| >2 pregnancies | 926 | (51.19) | 1 |  |  | 1 |  |  |
| **Number of artemether-lumefantrine treatments given** | |  |  |  |  |  |  |  |
| 0 | 959 | (52.14) |  |  |  |  |  |  |
| 1 | 311 | (64.95) | 1.78 | (1.35,2.34) | <0.001 |  |  |  |
| 2 | 103 | (70.87) | 2.36 | (1.50,3.73) | <0.001 |  |  |  |
| 3+ (max 6) | 41 | (82.93) | 4.58 | (1.98,10.58) | <0.001 |  |  |  |
| Continuous |  |  | 1.61 | (1.37,1.89) | <0.001 | 1.45 | (1.22,1.71) | <0.001 |
| **Number of SP doses** |  |  |  |  |  |  |  |  |
| 1 | 127 | (73.23) |  |  |  |  |  |  |
| 2 | 588 | (69.56) |  |  |  |  |  |  |
| 3 | 395 | (49.37) |  |  |  |  |  |  |
| 4 | 248 | (38.31) |  |  |  |  |  |  |
| 5 | 50 | (30) |  |  |  |  |  |  |
| 6 | 6 | (33.33) |  |  |  |  |  |  |
| Continuous |  |  | 0.553192 | (0.49,0.62) | <0.001 | 0.54 | (0.48,0.61) | <0.001 |

1. Active infection (acute and chronic)

| **Active infection (Acute or chronic)** | N | **% positive** | **Unadjusted Odds ratio** | **(95% CI)** | **P-value** | **Adjusted** | **(95% CI)** | **p-value** |
| --- | --- | --- | --- | --- | --- | --- | --- | --- |
| **Arm** |  |  |  |  |  |  |  |  |
| Intervention | 716 | (6.42) | 0.86 | (0.53,1.39) | 0.526 | 0.81 | (0.50,1.31) | 0.381 |
| Control | 698 | (7.45) | 1 |  |  | 1 |  |  |
| **Seasonality** |  |  |  |  |  |  |  |  |
| Born in rain reason (July-December) | 615 | (11.71) | 3.98 | (2.50,6.33) | <0.001 | 3.54 | (2.20,5.68) | <0.001 |
| Born in non-rain season (Jan-June) | 795 | (3.27) | 1 |  |  | 1 |  |  |
| **Gravida** |  |  |  |  |  |  |  |  |
| First or second pregnancy | 488 | (9.02) | 1.63 | (1.07,2.48) | 0.022 | 1.39 | (0.90,2.17) | 0.141 |
| >2 pregnancies | 926 | (5.83) | 1 |  |  | 1 |  |  |
| **Number of artemether-lumefantrine treatments given** | |  |  |  |  |  |  |  |
| 0 | 959 | (5.63) |  |  |  |  |  |  |
| 1 | 311 | (8.36) | 1.56 | (0.96,2.56) | 0.075 |  |  |  |
| 2 | 103 | (11.65) | 2.22 | (1.14,4.34) | 0.019 |  |  |  |
| 3+ (max 6) | 41 | (14.63) | 2.71 | (1.08,6.83) | 0.035 |  |  |  |
| Continuous |  |  | 1.39 | (1.13,1.72) | 0.002 | 1.30 | (1.03,1.64) | 0.025 |
| **Number of SP doses** |  |  |  |  |  |  |  |  |
| 1 | 127 | (11.81) |  |  |  |  |  |  |
| 2 | 588 | (11.05) |  |  |  |  |  |  |
| 3 | 395 | (3.8) |  |  |  |  |  |  |
| 4 | 248 | (0.81) |  |  |  |  |  |  |
| 5 | 50 | (0) |  |  |  |  |  |  |
| 6 | 6 | (16.67) |  |  |  |  |  |  |
| Continuous |  |  | 0.47 | (0.36,0.61) | <0.001 | 0.52 | (0.40,0.68) | <0.001 |

1. Past or Chronic infection

| **Past or Chronic infection** | N | **% positive** | **Unadjusted Odds ratio** | **(95% CI)** | **P-value** | **Adjusted** | **(95% CI)** | **p-value** |
| --- | --- | --- | --- | --- | --- | --- | --- | --- |
| **Arm** |  |  |  |  |  |  |  |  |
| Intervention | 716 | (54.75) | 0.93 | (0.66,1.31) | 0.685 | 0.94 | (0.67,1.33) | 0.744 |
| Control | 698 | (56.59) | 1 |  |  | 1 |  |  |
| **Seasonality** |  |  |  |  |  |  |  |  |
| Born in rain reason (July-December) | 615 | (48.94) | 0.64 | (0.51,0.79) | <0.001 | 0.50 | (0.40,0.64) | <0.001 |
| Born in non-rain season (Jan-June) | 795 | (60.63) | 1 |  |  | 1 |  |  |
| **Gravida** |  |  |  |  |  |  |  |  |
| First or second pregnancy | 488 | (68.03) | 2.22 | (1.76,2.82) | <0.001 | 2.23 | (1.73,2.88) | <0.001 |
| >2 pregnancies | 926 | (49.14) | 1 |  |  | 1 |  |  |
| **Number of artemether-lumefantrine treatments given** | |  |  |  |  |  |  |  |
| 0 | 959 | (50.57) |  |  |  |  |  |  |
| 1 | 311 | (63.34) | 1.75 | (1.33,2.30) | <0.001 |  |  |  |
| 2 | 103 | (69.9) | 2.38 | (1.51,3.74) | <0.001 |  |  |  |
| 3+ (max 6) | 41 | (80.49) | 4.16 | (1.87,9.23) | <0.001 |  |  |  |
| Continuous |  |  | 1.58 | (1.35,1.85) | <0.001 | 1.41 | (1.20,1.67) | <0.001 |
| **Number of SP doses** |  |  |  |  |  |  |  |  |
| 1 | 127 | (73.23) |  |  |  |  |  |  |
| 2 | 588 | (67.01) |  |  |  |  |  |  |
| 3 | 395 | (47.85) |  |  |  |  |  |  |
| 4 | 248 | (37.9) |  |  |  |  |  |  |
| 5 | 50 | (30) |  |  |  |  |  |  |
| 6 | 6 | (33.33) |  |  |  |  |  |  |
| Continuous |  |  | 0.57 | (0.50,0.64) | <0.001 | 0.55 | (0.49,0.62) | <0.001 |

1. Acute infection (malaria parasites ONLY - biopsy data)

| **ACUTE infection (biopsy, n=22)** | N | **% positive** | **Unadjusted Odds ratio** | **(95% CI)** | **P-value** | **Adjusted** | **(95% CI)** | **p-value** |
| --- | --- | --- | --- | --- | --- | --- | --- | --- |
| **Arm** |  |  |  |  |  |  |  |  |
| Intervention | 716 | (1.26) | 0.67 | (0.28,1.58) | 0.361 | 0.65 | (0.27,1.55) | 0.333 |
| Control | 698 | (1.86) | 1 |  |  | 1 |  |  |
| **Seasonality** |  |  |  |  |  |  |  |  |
| Born in rain reason (July-December) | 615 | (2.28) | 2.29 | (0.96,5.50) | 0.063 | 2.25 | (0.93,5.45) | 0.071 |
| Born in non-rain season (Jan-June) | 795 | (1.01) | 1 |  |  | 1 |  |  |
| **Gravida** |  |  |  |  |  |  |  |  |
| First or second pregnancy | 488 | (0.61) | 0.30 | (0.09,1.00) | 0.051 | 0.27 | (0.08,0.92) | 0.037 |
| >2 pregnancies | 926 | (2.05) | 1 |  |  | 1 |  |  |
| **Number of artemether-lumefantrine treatments given** | |  |  |  |  |  |  |  |
| 0 | 959 | (1.56) |  |  |  |  |  |  |
| 1 | 311 | (1.61) | 1.03 | (0.37,2.85) | 0.957 |  |  |  |
| 2 | 103 | (0.97) | 0.62 | (0.08,4.72) | 0.642 |  |  |  |
| 3+ (max 6) | 41 | (2.44) | 1.57 | (0.20,12.21) | 0.665 |  |  |  |
| Continous |  |  | 1.06 | (0.64,1.76) | 0.824 | 1.16 | (0.69,1.97) | 0.578 |
| **Number of SP doses** |  |  |  |  |  |  |  |  |
| 1 | 127 | (0) |  |  |  |  |  |  |
| 2 | 588 | (2.55) |  |  |  |  |  |  |
| 3 | 395 | (1.52) |  |  |  |  |  |  |
| 4 | 248 | (0.4) |  |  |  |  |  |  |
| 5 | 50 | (0) |  |  |  |  |  |  |
| 6 | 6 | (0) |  |  |  |  |  |  |
| At least 4 doses |  |  | 0.17 | (0.02,1.28) | 0.085 | 0.76 | (0.48,1.20) | 0.236 |

1. **Maternal peripheral infection at time of delivery (PCR)**

| **Maternal peripheral infection (PCR)** | N | **% positive** | **Unadjusted Odds ratio** | **(95% CI)** | **P-value** | **Adjusted** | **(95% CI)** | **p-value** |
| --- | --- | --- | --- | --- | --- | --- | --- | --- |
| **Arm** |  |  |  |  |  |  |  |  |
| Intervention | 830 | (17.83) | 1.02 | (0.79,1.32) | 0.875 | 0.98 | (0.75,1.28) | 0.867 |
| Control | 827 | (17.53) | 1 |  |  | 1 |  |  |
| **Seasonality** |  |  |  |  |  |  |  |  |
| Born in rain reason (July-December) | 762 | (28.08) | 4.03 | (3.05,5.34) | <0.001 | 4.04 | (3.04,5.36) | <0.001 |
| Born in non-rain season (Jan-June) | 895 | (8.83) | 1 |  |  | 1 |  |  |
| **Gravida** |  |  |  |  |  |  |  |  |
| First or second pregnancy | 581 | (21.17) | 1.44 | (1.11,1.87) | <0.001 | 1.31 | (0.99,1.72) | 0.056 |
| >2 pregnancies | 1,076 | (15.8) | 1 |  |  | 1 |  |  |
| **Number of artemether-lumefantrine treatments given** | |  |  |  |  |  |  |  |
| 0 | 1,148 | (15.59) |  |  |  |  |  |  |
| 1 | 350 | (20.57) | 1.40 | (1.03,1.90) | <0.001 |  |  |  |
| 2 | 114 | (24.56) | 1.76 | (1.12,2.78) | <0.001 |  |  |  |
| 3+ (max 6) | 45 | (31.11) | 2.45 | (1.27,4.70) | <0.001 |  |  |  |
| Continuous |  |  | 1.32 | (1.14,1.52) | <0.001 | 1.29 | (1.10,1.51) | 0.002 |
| **Number of SP doses** |  |  |  |  |  |  |  |  |
| 1 | 151 | (27.15) |  |  |  |  |  |  |
| 2 | 665 | (22.11) |  |  |  |  |  |  |
| 3 | 471 | (10.62) |  |  |  |  |  |  |
| 4 | 295 | (15.25) |  |  |  |  |  |  |
| 5 | 67 | (13.43) |  |  |  |  |  |  |
| 6 | 8 | (12.5) |  |  |  |  |  |  |
| Continuous |  |  | 0.74 | (0.64,0.84) | <0.001 | 0.78 | (0.68,0.89) | <0.001 |

**S3b: The Gambia**

1. Any malaria infection (active, chronic or both)

| **Any infection (acute, chronic or past)** | N | **% positive** | **Unadjusted Odds ratio** | **(95% CI)** | **P-value** | **Adjusted** | **(95% CI)** | **p-value** |
| --- | --- | --- | --- | --- | --- | --- | --- | --- |
| **Arm** |  |  |  |  |  |  |  |  |
| Intervention | 907 | (14.66) | 1.36 | (0.81,2.28) | 0.24 | 1.21 | (0.72,2.05) | 0.467 |
| Control | 850 | (10.47) | 1 |  |  | 1 |  |  |
| **Seasonality** |  |  |  |  |  |  |  |  |
| Born in rain reason (July-December) | 1,070 | (11.68) | 0.76 | (0.57,1.02) | 0.067 | 0.76 | (0.56,1.03) | 0.073 |
| Born in non-rain season (Jan-June) | 686 | (14.14) | 1 |  |  | 1 |  |  |
| **Gravida** |  |  |  |  |  |  |  |  |
| First or second pregnancy | 637 | (18.37) | 2.24 | (1.67,3.01) | <0.001 | 2.20 | (1.63,2.96) | <0.001 |
| >2 pregnancies | 1,118 | (9.39) | 1 |  |  | 1 |  |  |
| **Number of artemether-lumefantrine treatments given** |  |  |  |  |  |  |  |  |
| None | 1,699 | (11.71) | 1 |  |  | 1 |  |  |
| at least once | 58 | (39.66) | 4.44 | (2.49,7.93) | <0.001 | 4.30 | (2.38,7.78) | <0.001 |
| **No of SP doses** |  |  |  |  |  |  |  |  |
| 0 | 9 | (11.11) |  |  |  |  |  |  |
| 1 | 373 | (13.4) |  |  |  |  |  |  |
| 2 | 1,307 | (12.47) |  |  |  |  |  |  |
| 4 | 3 | (0) |  |  |  |  |  |  |
| At least 2 doses of SP |  |  | 0.93 | (0.66,1.32) | 0.693 | 0.96 | (0.67,1.38) | 0.844 |

1. Active infection (acute or chronic)

| **Active infection (acute or chronic)** | N | **% positive** | **Unadjusted Odds ratio** | **(95% CI)** | **P-value** | **Adjusted** | **(95% CI)** | **p-value** |
| --- | --- | --- | --- | --- | --- | --- | --- | --- |
| **Arm** |  |  |  |  |  |  |  |  |
| Intervention | 907 | (2.09) | 1.49 | (0.72,3.10) | 0.28 | 1.19 | (0.56,2.56) | 0.651 |
| Control | 850 | (1.41) | 1 |  |  | 1 |  |  |
| **Seasonality** |  |  |  |  |  |  |  |  |
| Born in rain reason (July-December) | 1,070 | (2.8) | 19.76 | (2.69,145.28) | 0.003 | 19.13 | (2.60,140.98) | 0.004 |
| Born in non-rain season (Jan-June) | 686 | (0.15) | 1 |  |  | 1 |  |  |
| **Gravida** |  |  |  |  |  |  |  |  |
| First or second pregnancy | 637 | (2.35) | 1.66 | (0.82,3.38) | 0.162 | 1.54 | (0.74,3.19) | 0.248 |
| >2 pregnancies | 1,118 | (1.43) | 1 |  |  | 1 |  |  |
| **Number of artemether-lumefantrine treatments given** |  |  |  |  |  |  |  |  |
| None | 1,699 | (1.47) | 1 |  |  | 1 |  |  |
| at least once | 58 | (10.34) | 7.73 | (3.04,19.63) | <0.001 | 6.10 | (2.24,16.60) | <0.001 |
| **No of SP doses** |  |  |  |  |  |  |  |  |
| 0 | 9 | (0) |  |  |  |  |  |  |
| 1 | 373 | (2.68) |  |  |  |  |  |  |
| 2 | 1,307 | (1.61) |  |  |  |  |  |  |
| 4 | 3 | (0) |  |  |  |  |  |  |
| At least 2 SP doses |  |  | 0.58 | (0.27,1.24) | 0.157 | 0.71 | (0.32,1.57) | 0.402 |

1. Past or Chronic infection

| **Past or Chronic infection** | N | **% positive** | **Unadjusted Odds ratio** | **(95% CI)** | **P-value** | **Adjusted** | **(95% CI)** | **p-value** |
| --- | --- | --- | --- | --- | --- | --- | --- | --- |
| **Arm** |  |  |  |  |  |  |  |  |
| Intervention | 907 | (13.01) | 1.33 | (0.81,2.19) | 0.254 | 1.21 | (0.73,2.00) | 0.463 |
| Control | 850 | (9.41) | 1 |  |  | 1 |  |  |
| **Seasonality** |  |  |  |  |  |  |  |  |
| Born in rain reason (July-December) | 1,070 | (9.44) | 0.59 | (0.44,0.81) | 0.001 | 0.60 | (0.44,0.81) | 0.001 |
| Born in non-rain season (Jan-June) | 686 | (14.14) | 1 |  |  | 1 |  |  |
| **Gravida** |  |  |  |  |  |  |  |  |
| First or second pregnancy | 637 | (16.64) | 2.27 | (1.67,3.09) | <0.001 | 2.23 | (1.63,3.04) | <0.001 |
| >2 pregnancies | 1,118 | (8.23) | 1 |  |  | 1 |  |  |
| **Number of artemether-lumefantrine treatments given** |  |  |  |  |  |  |  |  |
| None | 1,699 | (10.59) | 1 |  |  | 1 |  |  |
| at least once | 58 | (31.03) | 3.26 | (1.78,5.98) | <0.001 | 3.19 | (1.71,5.95) | <0.001 |
| **No of SP doses** |  |  |  |  |  |  |  |  |
| 0 | 9 | (11.11) |  |  |  |  |  |  |
| 1 | 373 | (11.8) |  |  |  |  |  |  |
| 2 | 1,307 | (11.09) |  |  |  |  |  |  |
| 4 | 3 | (0) |  |  |  |  |  |  |
| At least 2 SP |  |  | 0.95 | (0.66,1.36) | 0.768 | 0.96 | (0.66,1.39) | 0.812 |

1. Peripheral infection at time of delivery (PCR)

| **Maternal peripheral infection (PCR)** | N | **% positive** | **Unadjusted Odds ratio** | **(95% CI)** | **P-value** | **Adjusted** | **(95% CI)** | **p-value** |
| --- | --- | --- | --- | --- | --- | --- | --- | --- |
| **Arm** |  |  |  |  |  |  |  |  |
| Intervention | 765 | (2.61) | 1.05 | (0.52,2.10) | 0.902 | 0.83 | (0.38,1.82) | 0.645 |
| Control | 737 | (2.58) | 1 |  |  | 1 |  |  |
| **Seasonality** |  |  |  |  |  |  |  |  |
| Born in rain reason (July-December) | 926 | (3.78) | 5.74 | (2.02,16.30) | 0.001 | 5.71 | (1.99,16.36) | 0.001 |
| Born in non-rain season (Jan-June) | 576 | (0.69) | 1 |  |  | 1 |  |  |
| **Gravida** |  |  |  |  |  |  |  |  |
| First or second pregnancy | 534 | (2.62) | 1.03 | (0.53,2.00) | 0.934 | 0.96 | (0.48,1.93) | 0.914 |
| >2 pregnancies | 967 | (2.59) | 1 |  |  | 1 |  |  |
| **Number of artemether-lumefantrine treatments given** |  |  |  |  |  |  |  |  |
| None | 1,457 | (2.2) | 1 |  |  | 1 |  |  |
| at least once | 45 | (15.56) | 8.49 | (3.45,20.90) | <0.001 | 8.64 | (3.28,22.77) | <0.001 |
| **No of SP doses** |  |  |  |  |  |  |  |  |
| 0 | 4 | (0) |  |  |  |  |  |  |
| 1 | 327 | (3.98) |  |  |  |  |  |  |
| 2 | 1,110 | (2.25) |  |  |  |  |  |  |
| 4 | 3 | (33.33) |  |  |  |  |  |  |
| At least 2 doses |  |  | 0.55 | (0.28,1.09) | 0.087 | 0.65 | (0.32,1.33) | 0.24 |

**S3c: Benin – Acute infection (by PCR) adjusted by all other variables in the tables.**

| **Active infection (PCR)** | N | **% positive** | **Unadjusted Odds ratio** | **(95% CI)** | **P-value** | **Adjusted** | **(95% CI)** | **p-value** |
| --- | --- | --- | --- | --- | --- | --- | --- | --- |
| **Arm** |  |  |  |  |  |  |  |  |
| Intervention | 357 | (11.2) | 0.79 | (0.49,1.28) | 0.343 | 0.75 | (0.45,1.25) | 0.266 |
| Control | 262 | (13.74) | 1 |  |  | 1 |  |  |
| **Seasonality** |  |  |  |  |  |  |  |  |
| Born in rain reason (Apr-jul, Oct-Nov) | 294 | (11.56) | 0.88 | (0.54,1.43) | 0.607 | 0.84 | (0.51,1.37) | 0.48 |
| Born in non-rain season (dec-mar, aug-sept) | 325 | (12.92) | 1 |  |  | 1 |  |  |
| **Gravida** |  |  |  |  |  |  |  |  |
| First or second pregnancy | 214 | (13.08) | 1.12 | (0.68,1.84) | 0.657 | 1.11 | (0.67,1.84) | 0.673 |
| >2 pregnancies | 405 | (11.85) | 1 |  |  | 1 |  |  |
| **Coartem** |  |  |  |  |  |  |  |  |
| None | 503 | (12.33) | 1.00 |  |  |  |  |  |
| at least once | 116 | (12.07) | 0.98 | (0.53,1.81) | 0.939 | 1.09 | (0.56,2.11) | 0.804 |
| **No of SP doses** |  |  |  |  |  |  |  |  |
| 1 | 199 | (14.57) |  |  |  |  |  |  |
| 2 | 418 | (11.24) |  |  |  |  |  |  |
| 3 | 2 | (0) |  |  |  |  |  |  |
| At least 2 doses |  |  | 0.74 | (0.45,1.21) | 0.232 | 0.70 | (0.42,1.16) | 0.165 |

**Table S4: Anaemia at delivery by country**

S4a: Burkina Faso

| **Anaemia (<11g/dL)** | N | **% anaemic** | **Unadjusted Odds ratio** | **(95% CI)** | **P-value** | **Adjusted** | **(95% CI)** | **p-value** |
| --- | --- | --- | --- | --- | --- | --- | --- | --- |
| **Arm** |  |  |  |  |  |  |  |  |
| Intervention | 854 | (25.64) | 1.10 | (0.78,1.55) | 0.576 | 1.09 | (0.77,1.54) | 0.626 |
| Control | 838 | (23.63) | 1 |  |  | 1 |  |  |
| **Seasonality** |  |  |  |  |  |  |  |  |
| Born in rain reason (July-December) | 791 | (29.08) | 1.55 | (1.24,1.95) | <0.001 | 1.54 | (1.23,1.94) | <0.001 |
| Born in non-rain season (Jan-June) | 901 | (20.75) | 1 |  |  | 1 |  |  |
| **Gravida** |  |  |  |  |  |  |  |  |
| First or second pregnancy | 600 | (25.67) | 1.07 | (0.85,1.36) | 0.554 | 1.03 | (0.81,1.32) | 0.782 |
| >2 pregnancies | 1,092 | (24.08) | 1 |  |  | 1 |  |  |
| **Number of AL treatment given** | |  |  |  |  |  |  |  |
| 0 | 1,172 | (24.32) |  |  |  |  |  |  |
| 1 | 361 | (22.71) | 0.91 | (0.68,1.21) | 0.502 |  |  |  |
| 2 | 115 | (33.04) | 1.53 | (1.00,2.33) | 0.048 |  |  |  |
| 3+ (max 6) | 44 | (27.27) | 1.24 | (0.62,2.48) | 0.544 |  |  |  |
| Continuous |  |  | 1.08 | (0.94,1.25) | 0.268 | 1.07 | (0.92,1.24) | 0.381 |
| **Number of SP doses** |  |  |  |  |  |  |  |  |
| 1 | 153 | (31.37) |  |  |  |  |  |  |
| 2 | 671 | (24.89) |  |  |  |  |  |  |
| 3 | 482 | (22.41) |  |  |  |  |  |  |
| 4 | 309 | (23.3) |  |  |  |  |  |  |
| 5 | 69 | (28.99) |  |  |  |  |  |  |
| 6 | 8 | (25) |  |  |  |  |  |  |
| Continuous |  |  | 0.915167 | (0.82,1.02) | 0.121 | 0.93 | (0.83,1.04) | 0.203 |

S4b: The Gambia

| **Anaemia (<11g/dL)** | N | **% positive** | **Unadjusted Odds ratio** | **(95% CI)** | **P-value** | **Adjusted** | **(95% CI)** | **p-value** |
| --- | --- | --- | --- | --- | --- | --- | --- | --- |
| **Arm** |  |  |  |  |  |  |  |  |
| Intervention | 937 | (63.93) | 1.11 | (0.92,1.34) | 0.28 | 1.15 | (0.95,1.39) | 0.162 |
| Control | 880 | (61.48) | 1 |  |  | 1 |  |  |
| **Seasonality** |  |  |  |  |  |  |  |  |
| Born in rain reason (July-December) | 1,092 | (65.11) | 1.29 | (1.06,1.56) | 0.01 | 1.29 | (1.06,1.57) | 0.01 |
| Born in non-rain season (Jan-June) | 725 | (59.17) | 1 |  |  | 1 |  |  |
| **Gravida** |  |  |  |  |  |  |  |  |
| First or second pregnancy | 658 | (58.97) | 0.78 | (0.64,0.95) | 0.012 | 0.78 | (0.64,0.95) | 0.014 |
| >2 pregnancies | 1,157 | (64.91) | 1 |  |  | 1 |  |  |
| **No of AL treatment given** |  |  |  |  |  |  |  |  |
| None | 1,759 | (62.93) | 1 |  |  | 1 |  |  |
| at least once | 58 | (56.9) | 0.78 | (0.46,1.32) | 0.35 | 0.73 | (0.43,1.25) | 0.248 |
| **No of SP doses** |  |  |  |  |  |  |  |  |
| 0 | 10 | (30) |  |  |  |  |  |  |
| 1 | 388 | (66.24) |  |  |  |  |  |  |
| 2 | 1,351 | (62.1) |  |  |  |  |  |  |
| 4 | 3 | (66.67) |  |  |  |  |  |  |
| At least 2 doses of SP |  |  | 0.87 | (0.69,1.09) | 0.228 | 0.87 | (0.68,1.09) | 0.228 |

S4c: Benin

| **Anaemia (<11g/dL)** | N | **% positive** | **Unadjusted Odds ratio** | **(95% CI)** | **P-value** | **Adjusted** | **(95% CI)** | **p-value** |
| --- | --- | --- | --- | --- | --- | --- | --- | --- |
| **Arm** |  |  |  |  |  |  |  |  |
| Intervention | 376 | (42.82) | 0.85 | (0.50,1.42) | 0.528 | 0.83 | (0.49,1.42) | 0.497 |
| Control | 271 | (45.02) | 1 |  |  | 1 |  |  |
| **Seasonality** |  |  |  |  |  |  |  |  |
| Born in rain reason (Apr-jul, Oct-Nov) | 311 | (47.59) | 1.39 | (1.00,1.92) | 0.048 | 1.35 | (0.97,1.88) | 0.073 |
| Born in non-rain season (dec-mar, aug-sept) | 336 | (40.18) | 1 |  |  | 1 |  |  |
| **Gravida** |  |  |  |  |  |  |  |  |
| First or second pregnancy | 222 | (41.44) | 0.85 | (0.61,1.20) | 0.355 | 0.84 | (0.60,1.19) | 0.33 |
| >2 pregnancies | 425 | (44.94) | 1 |  |  | 1 |  |  |
| **No AL treatment given** |  |  |  |  |  |  |  |  |
| None | 527 | (43.83) | 1.00 |  |  |  |  |  |
| at least once | 120 | (43.33) | 0.97 | (0.63,1.50) | 0.908 | 1.05 | (0.67,1.65) | 0.829 |
| **No of SP doses** |  |  |  |  |  |  |  |  |
| 1 | 213 | (47.89) |  |  |  |  |  |  |
| 2 | 432 | (41.9) |  |  |  |  |  |  |
| 3 | 2 | (0) |  |  |  |  |  |  |
| At least 2 doses |  |  | 0.78 | (0.55,1.10) | 0.153 | 0.80 | (0.56,1.14) | 0.213 |

**Table S5: Lowbirthweight by country**

*Birthweight excludes miscarriage, stillbirths and twins.

S5a: Burkina Faso

| **Low birthweight (<2.5kg)** | N | **% lowbwt** | **Unadjusted Odds ratio** | **(95% CI)** | **P-value** | **Adjusted** | **(95% CI)** | **p-value** |
| --- | --- | --- | --- | --- | --- | --- | --- | --- |
| **Arm** |  |  |  |  |  |  |  |  |
| Intervention | 837 | (10.63) | 0.97 | (0.61,1.55) | 0.907 | 1.01 | (0.63,1.63) | 0.964 |
| Control | 826 | (10.53) | 1 |  |  | 1 |  |  |
| **Seasonality** |  |  |  |  |  |  |  |  |
| Born in rain reason (July-December) | 784 | (12.24) | 1.43 | (1.04,1.97) | 0.028 | 1.38 | (1.00,1.92) | 0.052 |
| Born in non-rain season (Jan-June) | 879 | (9.1) | 1 |  |  | 1 |  |  |
| **Gravida** |  |  |  |  |  |  |  |  |
| First or second pregnancy | 584 | (16.78) | 2.72 | (1.97,3.77) | <0.001 | 2.68 | (1.92,3.74) | <0.001 |
| >2 pregnancies | 1,079 | (7.23) | 1 |  |  | 1 |  |  |
| **Number of AL treatment given** | |  |  |  |  |  |  |  |
| 0 | 1,151 | (9.82) |  |  |  |  |  |  |
| 1 | 356 | (11.52) | 1.21 | (0.82,1.78) | 0.344 |  |  |  |
| 2 | 112 | (12.5) | 1.27 | (0.69,2.33) | 0.435 |  |  |  |
| 3+ (max 6) | 44 | (18.18) | 1.89 | (0.84,4.26) | 0.123 |  |  |  |
| Continuous |  |  | 1.18 | (0.98,1.42) | 0.083 | 1.02 | (0.84,1.25) | 0.82 |
| **Number of SP doses** |  |  |  |  |  |  |  |  |
| 1 | 148 | (21.62) |  |  |  |  |  |  |
| 2 | 663 | (11.01) |  |  |  |  |  |  |
| 3 | 473 | (9.3) |  |  |  |  |  |  |
| 4 | 302 | (6.62) |  |  |  |  |  |  |
| 5 | 69 | (10.14) |  |  |  |  |  |  |
| 6 | 8 | (0) |  |  |  |  |  |  |
| Continuous |  |  | 0.735469 | (0.62,0.87) | <0.001 | 0.75 | (0.63,0.88) | 0.001 |

S5b: Gambia

| **Low birthweight** | N | **% positive** | **Unadjusted Odds ratio** | **(95% CI)** | **P-value** | **Adjusted** | **(95% CI)** | **p-value** |
| --- | --- | --- | --- | --- | --- | --- | --- | --- |
| **Arm** |  |  |  |  |  |  |  |  |
| Intervention | 911 | (9.88) | 1.03 | (0.65,1.63) | 0.912 | 0.96 | (0.59,1.55) | 0.852 |
| Control | 855 | (10.64) | 1 |  |  | 1 |  |  |
| **Seasonality** |  |  |  |  |  |  |  |  |
| Born in rain reason (July-December) | 1,066 | (8.44) | 0.64 | (0.47,0.87) | 0.005 | 0.62 | (0.45,0.85) | 0.003 |
| Born in non-rain season (Jan-June) | 700 | (13) | 1 |  |  | 1 |  |  |
| **Gravida** |  |  |  |  |  |  |  |  |
| First or second pregnancy | 645 | (14.11) | 1.94 | (1.42,2.66) | <0.001 | 1.91 | (1.39,2.63) | <0.001 |
| >2 pregnancies | 1,119 | (8.04) | 1 |  |  | 1 |  |  |
| **No of AL treatment given** |  |  |  |  |  |  |  |  |
| None | 1,713 | (9.87) | 1 |  |  | 1 |  |  |
| at least once | 53 | (22.64) | 2.92 | (1.47,5.80) | 0.002 | 2.68 | (1.32,5.43) | 0.006 |
| **No of SP doses** |  |  |  |  |  |  |  |  |
| 0 | 10 | (20) |  |  |  |  |  |  |
| 1 | 367 | (13.9) |  |  |  |  |  |  |
| 2 | 1,321 | (9.24) |  |  |  |  |  |  |
| 4 | 3 | (33.33) |  |  |  |  |  |  |
| At least 2 doses of SP |  |  | 0.59 | (0.42,0.84) | 0.003 | 0.59 | (0.41,0.85) | 0.004 |

S5c: Benin

| **Low birthweight** | N | **% positive** | **Unadjusted Odds ratio** | **(95% CI)** | **P-value** | **Adjusted** | **(95% CI)** | **p-value** |
| --- | --- | --- | --- | --- | --- | --- | --- | --- |
| **Arm** |  |  |  |  |  |  |  |  |
| Intervention | 379 | (10.55) | 1.21 | (0.60,2.44) | 0.598 | 1.17 | (0.56,2.44) | 0.683 |
| Control | 269 | (8.55) | 1 |  |  | 1 |  |  |
| **Seasonality** |  |  |  |  |  |  |  |  |
| Born in rain reason (Apr-jul, Oct-Nov) | 309 | (8.74) | 0.78 | (0.46,1.33) | 0.361 | 0.74 | (0.43,1.26) | 0.265 |
| Born in non-rain season (dec-mar, aug-sept) | 339 | (10.62) | 1 |  |  | 1 |  |  |
| **Gravida** |  |  |  |  |  |  |  |  |
| First or second pregnancy | 227 | (10.13) | 1.05 | (0.61,1.83) | 0.853 | 1.04 | (0.60,1.82) | 0.88 |
| >2 pregnancies | 421 | (9.5) | 1 |  |  | 1 |  |  |
| **Coartem** |  |  |  |  |  |  |  |  |
| None | 529 | (9.83) | 1.00 |  |  |  |  |  |
| at least once | 119 | (9.24) | 0.93 | (0.46,1.89) | 0.844 | 0.92 | (0.44,1.92) | 0.822 |
| **No of SP doses** |  |  |  |  |  |  |  |  |
| 1 | 216 | (12.96) |  |  |  |  |  |  |
| 2 | 431 | (8.12) |  |  |  |  |  |  |
| 3 | 1 | (0) |  |  |  |  |  |  |
| At least 2 doses |  |  | 0.58 | (0.34,1.00) | 0.05 | 0.57 | (0.33,0.98) | 0.043 |

**Table S6: Adverse pregnancy outcomes by country**

S6a: Burkina Faso

|  | **Control** |  | **Intervention** |  |  |  |  |
| --- | --- | --- | --- | --- | --- | --- | --- |
|  | **N** | **%** | **N** | **%** | **Odds ratio** | **(95% CI)** | **p-value** |
| **Adverse outcomes** |  |  |  |  |  |  |  |
| Congenital abnomalities | 7 | (0.81) | 14 | (1.61) | 1.99 | (0.76,5.26) | 0.164 |
| **Miscarriage | 2 | (0.22) | 9 | (1) | 4.54 | (0.98,21.05) | 0.054 |
| Preterm birth | 30 | (3.46) | 18 | (2.06) | 0.55 | (0.22,1.38) | 0.201 |
| Stillbirth | 14 | (1.62) | 11 | (1.26) | 0.78 | (0.35,1.72) | 0.535 |
| Adverse: miscarriage, preterm or stillbirth | 39 | (4.33) | 35 | (3.89) | 0.90 | (0.51,1.59) | 0.72 |
| **Deaths** |  |  |  |  |  |  |  |
| Perinatal death | 5 | (0.58) | 3 | (0.34) | 0.59 | (0.14,2.50) | 0.477 |
| **Maternal death | 0 | (0) | 1 | (0.11) | - | - | - |
| Adverse: Perinatal death, miscarriage, preterm or stillbirth | 40 | (4.44) | 37 | (4.11) | 0.93 | (0.54,1.60) | 0.788 |

**Denominator is all enrolled as outcome can occur anytime during pregnancy. All other variables, denominator is total women who delivered in study.

S6b: The Gambia

|  | **Control** |  | **Intervention** | | **Odds ratio** | **(95% CI)** | **p value** |
| --- | --- | --- | --- | --- | --- | --- | --- |
|  | **N** | **%** | **N** | **%** | **/mean diff** | |  |
| **Adverse outcomes** |  |  |  |  |  |  |  |
| Congenital abnormalities | 7 | (0.79) | 12 | (1.26) | 1.61 | (0.63,4.09) | 0.322 |
| ***Miscarriage | 12 | (1.26) | 6 | (0.6) | 0.43 | (0.12,1.54) | 0.194 |
| Preterm birth | 22 | (2.47) | 39 | (4.08) | 1.61 | (0.82,3.19) | 0.169 |
| **Stillbirth | 21 | (2.35) | 30 | (3.14) | 1.26 | (0.65,2.44) | 0.501 |
| Adverse totals: miscarriage, preterm or stillbirth | 47 | (4.94) | 63 | (6.25) | 1.24 | (0.79,1.94) | 0.355 |
| **Deaths** |  |  |  |  |  |  |  |
| Perinatal death | 13 | (1.46) | 15 | (1.57) | 1.08 | (0.51,2.28) | 0.842 |
| ***Maternal death | 5 | (0.53) | 2 | (0.2) | 0.38 | (0.07,1.95) | 0.244 |
| Adverse totals: Perinatal deaths, miscarriage, preterm or stillbirth | 60 | (6.3) | 76 | (7.54) | 1.18 | (0.78,1.76) | 0.432 |

**2 extra stillbirths reported at time of delivery but not recorded as SAEs, ***Denominator is all enrolled as outcome can occur anytime during pregnancy. All other variables, denominator is total women who delivered in study.

S6c: Benin

|  | **Control** |  | **Intervention** |  | **Odds ratio /** |  |  |
| --- | --- | --- | --- | --- | --- | --- | --- |
|  | **N** | **%** | **N** | **%** | **mean diff** | **(95% CI)** | **p-value** |
| **Adverse outcomes** |  |  |  |  |  |  |  |
| Congenital abnormalities | 2 | (0.7) | 3 | (0.76) | 1.07 | (0.16,7.26) | 0.946 |
| **Miscarriage | 2 | (0.47) | 1 | (0.18) | 0.39 | (0.04,4.37) | 0.448 |
| Preterm birth | 8 | (2.8) | 13 | (3.27) | 1.18 | (0.48,2.88) | 0.722 |
| Stillbirth | 4 | (1.4) | 6 | (1.51) | 1.03 | (0.18,5.72) | 0.974 |
| Adverse totals: miscarriage, preterm or stillbirth | 13 | (3.02) | 18 | (3.31) | 1.10 | (0.53,2.28) | 0.797 |
| **Deaths** |  |  |  |  |  |  |  |
| Perinatal death | 3 | (1.05) | 6 | (1.51) | 1.49 | (0.34,6.51) | 0.593 |
| **Maternal death | 1 | (0.23) | 0 | (0) | - | - | - |
| Adverse totals: Perinatal deaths, miscarriage, preterm or stillbirth | 14 | (3.26) | 18 | (3.31) | 1.02 | (0.50,2.07) | 0.959 |

** Denominator is all enrolled as outcome can occur anytime during pregnancy. All other variables, denominator is total women who delivered in study.

**Table S7: Effect of the intervention on antenatal clinic attendance and IPTp-SP coverage by country.**

S7a: Burkina Faso

|  | **Control** |  | **Intervention** | | **Odds ratio / IRR** | **(95% CI)** | **p-value** |
| --- | --- | --- | --- | --- | --- | --- | --- |
|  | **N** | **%** | **N** | **%** |  |  |  |
| **ANC visits** |  |  |  |  |  |  |  |
| **Woman's level** |  |  |  |  |  |  |  |
| 1 schedule visit | 65 | (7.22) | 46 | (5.11) |  |  |  |
| 2 schedule visits | 164 | (18.22) | 95 | (10.56) |  |  |  |
| 3 schedule visits | 292 | (32.44) | 277 | (30.78) |  |  |  |
| 4 schedule visits | 262 | (29.11) | 325 | (36.11) |  |  |  |
| 5 schedule visits | 100 | (11.11) | 142 | (15.78) |  |  |  |
| 6 schedule visits | 15 | (1.67) | 15 | (1.67) |  |  |  |
| 7 schedule visits | 2 | (0.22) | 0 | (0) |  |  |  |
| At least 2 schedule visits | 835 | (92.78) | 854 | (94.89) | 1.43 | (0.85, 2.38) | 0.175 |
| At least 4 schedule visits | 379 | (42.11) | 482 | (53.56) | 1.62 | (1.02,2.59) | 0.041 |
| **At village level** |  |  |  |  |  |  |  |
| Mean number of schedule visits (SD) | 3.24 | (0.46) | 3.51 | (0.32) | 1.08 | (1.00,1.17) | 0.045 |
| Mean number of unschedule visits (SD) | 0.62 | (0.32) | 0.56 | (0.31) | 0.90 | (0.63,1.30) | 0.589 |
| Mean number of any ANC visits (SD) | 3.87 | (0.56) | 4.08 | (0.52) | 1.06 | (0.96,1.16) | 0.258 |
|  |  |  |  |  |  |  |  |
| **IPTp-SP coverage** |  |  |  |  |  |  |  |
| **Woman's level** |  |  |  |  |  |  |  |
| 1 dose | 114 | (12.67) | 91 | (10.11) |  |  |  |
| 2 doses | 364 | (40.44) | 345 | (38.33) |  |  |  |
| 3 doses | 237 | (26.33) | 255 | (28.33) |  |  |  |
| 4 doses | 145 | (16.11) | 170 | (18.89) |  |  |  |
| 5 doses | 33 | (3.67) | 38 | (4.22) |  |  |  |
| 6 doses | 7 | (0.78) | 1 | (0.11) |  |  |  |
| **At village level** |  |  |  |  |  |  |  |
| Mean number of SP doses | 2.60 | (0.22) | 2.69 | (0.27) | 1.04 | (0.97,1.10) | 0.294 |
| At least 2 doses of SP |  |  |  |  |  |  |  |
| No | 114 | (12.67) | 91 | (10.11) |  |  |  |
| Yes | 786 | (87.33) | 809 | (89.89) | 1.29 | (0.96,1.73) | 0.093 |
| At least 4 doses of SP |  |  |  |  |  |  |  |
| No | 715 | (79.44) | 691 | (76.78) |  |  |  |
| Yes | 185 | (20.56) | 209 | (23.22) | 1.14 | (0.76,1.72) | 0.517 |

S7b: The Gambia

|  | **Control** |  | **Intervention** | | **Odd ratio/ IRR** | **(95% CI)** | **P value** |
| --- | --- | --- | --- | --- | --- | --- | --- |
|  | **N** | **%** | **N** | **%** |  |  |  |
| **ANC visits** |  |  |  |  |  |  |  |
| **Woman's level** |  |  |  |  |  |  |  |
| 1 schedule visit | 201 | (21.11) | 197 | (19.54) |  |  |  |
| 2 schedule visits | 620 | (65.13) | 674 | (66.87) |  |  |  |
| 3 schedule visits | 109 | (11.45) | 118 | (11.71) |  |  |  |
| 4 schedule visits | 20 | (2.1) | 18 | (1.79) |  |  |  |
| 5 schedule visits | 2 | (0.21) | 1 | (0.1) |  |  |  |
| At least 2 schedule visits | 751 | (78.89) | 811 | (80.46) | 1.03 | (0.66,1.62) | 0.881 |
| **At village level** |  |  |  |  |  |  |  |
| Mean number of schedule visits (SD) | 1.96 | (0.25) | 1.94 | (0.19) | 1.004 | (0.93,1.09) | 0.912 |
| Mean number of unscheduled visits (SD) | 0.48 | (0.31) | 0.55 | (0.39) | 1.335 | (0.80,2.22) | 0.266 |
| Mean number of any ANC visits (SD) | 2.43 | (0.51) | 2.48 | (0.57) | 1.069 | (0.90,1.27) | 0.445 |
|  |  |  |  |  |  |  |  |
| **IPTp-SP coverage** |  |  |  |  |  |  |  |
| **Woman's level** |  |  |  |  |  |  |  |
| 0 doses | 4 | (0.42) | 8 | (0.79) |  |  |  |
| 1 dose | 231 | (24.26) | 232 | (23.02) |  |  |  |
| 2 doses | 688 | (72.27) | 724 | (71.83) |  |  |  |
| 3 doses | 28 | (2.94) | 42 | (4.17) |  |  |  |
| 4 doses | 1 | (0.11) | 2 | (0.2) |  |  |  |
| **At village level** |  |  |  |  |  |  |  |
| Mean number of SP doses | 1.80 | (0.13) | 1.79 | (0.12) | 1.011 | (0.97,1.05) | 0.611 |
| At least 2 doses of SP |  |  |  |  |  |  |  |
| No | 235 | (24.68) | 240 | (23.81) |  |  |  |
| Yes | 717 | (75.32) | 768 | (76.19) | 1.01 | (0.70,1.46) | 0.938 |

S7c: Benin

|  | **Control** |  | **Intervention** | | **Odds ratio** | **(95% CI)** | **p-value** |
| --- | --- | --- | --- | --- | --- | --- | --- |
|  | **N** | **%** | **N** | **%** | **/IRR** |  |  |
| **ANC visits** |  |  |  |  |  |  |  |
| **Woman's level** |  |  |  |  |  |  |  |
| **Schedule visits** |  |  |  |  |  |  |  |
| 1 schedule visit | 180 | (41.96) | 244 | (45.02) |  |  |  |
| 2 schedule visits | 249 | (58.04) | 298 | (54.98) | 0.88 | (0.63,1.24) | 0.471 |
| **Unscheduled visits** |  |  |  |  |  |  |  |
| 0 unscheduled visits | 375 | (87.41) | 472 | (87.08) |  |  |  |
| 1 unscheduled visit | 47 | (10.96) | 59 | (10.89) |  |  |  |
| 2 unscheduled visits | 6 | (1.4) | 8 | (1.48) |  |  |  |
| 3 unscheduled visits | 1 | (0.23) | 3 | (0.55) |  |  |  |
| **At village level** |  |  |  |  |  |  |  |
| Mean number of schedule visits (SD) | 1.57 | (0.19) | 1.56 | (0.10) | 0.981 | (0.93,1.04) | 0.484 |
| Mean number of unscheduled visits (SD) | 0.15 | (0.10) | 0.17 | (0.15) | 1.072 | (0.61,1.89) | 0.810 |
| Mean number of any ANC visits (SD) | 1.72 | (0.23) | 1.73 | (0.21) | 0.988 | (0.91,1.07) | 0.781 |
| **IPTp-SP coverage** |  |  |  |  |  |  |  |
| **Woman's level** |  |  |  |  |  |  |  |
| 1 dose | 180 | (41.96) | 246 | (45.39) |  |  |  |
| 2 doses | 247 | (57.58) | 296 | (54.61) |  |  |  |
| 3 doses | 2 | (0.47) | 0 | (0) |  |  |  |
| **At village level** |  |  |  |  |  |  |  |
| Mean number of SP doses | 1.58 | (0.20) | 1.55 | (0.11) | 0.975 | (0.92,1.03) | 0.387 |
| At least 2 doses of SP |  |  |  |  |  |  |  |
| No | 180 | (41.96) | 246 | (45.39) |  |  |  |
| Yes | 249 | (58.04) | 296 | (54.61) | 0.87 | (0.62,1.22) | 0.418 |

**Table S8: Serious adverse events (SAE) by trial arm and country**

**A1) Burkina Faso:** reported SAEs by main cause and outcome

|  |  |  | Outcome |  |  |  |
| --- | --- | --- | --- | --- | --- | --- |
| Cause | Recovered | Maternal  death | Perinatal  death | Spon. Abortion | Stillbirth | Total |
| malaria | 3 | 0 | 1 | 4 | 0 | 8 |
| anaemia | 2 | 0 | 0 | 0 | 0 | 2 |
| birth asphyxia | 0 | 0 | 0 | 0 | 1 | 1 |
| pre-eclampsia | 1 | 0 | 0 | 0 | 0 | 1 |
| stillbirth | 0 | 0 | 0 | 0 | 13 | 13 |
| spon abortion | 0 | 0 | 0 | 4 | 0 | 4 |
| preterm | 0 | 0 | 4 | 0 | 0 | 4 |
| congenital abnormalities | 5 | 0 | 2 | 0 | 3 | 10 |
| other | 5 | 1 | 1 | 2 | 8 | 17 |
| Total | 16 | 1 | 8 | 10 | 25 | 60 |

Other includes: urinary tract infection, trauma, placental retention, cord prolapse, dystocia, chorioamniotitis, haemorrhage, premature rupture of membrane, circular of cord. For maternal death other, cause was uterine rupture. For Perinatal death, cause was mechanical dystocia

A2) BURKINA FASO: Main cause of SAEs by study arm

|  | **Control** |  | **Intervention** | |
| --- | --- | --- | --- | --- |
| **Main cause of SAE** | **N** | **%** | **N** | **%** |
| malaria | 3 | (10.71) | 5 | (15.63) |
| anaemia | 1 | (3.57) | 1 | (3.13) |
| birth asphyxia | 1 | (3.57) | 0 | (0) |
| pre-eclampsia | 1 | (3.57) | 0 | (0) |
| stillbirth | 5 | (17.86) | 8 | (25) |
| Spon. abortion | 1 | (3.57) | 3 | (9.38) |
| preterm | 2 | (7.14) | 2 | (6.25) |
| Other | 8 | (28.57) | 9 | (28.13) |
| congenital abnormalities | 6 | (21.43) | 4 | (12.5) |
| Total | 28 |  | 32 |  |

**B1) Gambia:** reported SAEs by main cause and outcome

|  |  |  |  | Outcome |  |  |  |
| --- | --- | --- | --- | --- | --- | --- | --- |
| Main Cause | Alive/recovered | Congenital abnormalities | Maternal death | Perinatal death | Spontaneous abortion | Stillbirth | Total |
| Abruptio placenta | 1 | 0 | 2 | 0 | 0 | 10 | 13 |
| Anaemia | 6 | 0 | 0 | 0 | 0 | 0 | 6 |
| birth asphyxia | 0 | 0 | 0 | 16 | 0 | 0 | 16 |
| Congenital abnormalities | 0 | 0 | 0 | 1 | 0 | 0 | 1 |
| malaria | 1 | 0 | 0 | 0 | 1 | 0 | 2 |
| malaria and pre-eclampsia/hypertension | 0 | 0 | 0 | 0 | 0 | 1 | 1 |
| neonatal sepsis | 2 | 0 | 0 | 10 | 0 | 0 | 12 |
| placental Previa | 1 | 0 | 0 | 0 | 1 | 2 | 4 |
| pre-eclampsia/eclampsia | 11 | 0 | 0 | 0 | 0 | 3 | 14 |
| prolonged labour | 1 | 0 | 0 | 0 | 0 | 0 | 1 |
| spontaneous abortion | 2 | 0 | 0 | 0 | 4 | 0 | 6 |
| stillbirth | 0 | 0 | 0 | 0 | 0 | 30 | 30 |
| other | 10 | 1 | 5 | 1 | 1 | 3 | 21 |
| Total | 35 | 1 | 7 | 28 | 7 | 49 | 127 |

Other includes: For maternal deaths these are: postpartum sepsis, postpartum hemorrhage, severe hyper emesis, diarrhoea and suspected case of embolism, meningitis and 1 due to complication post operative of C-section. One Perinatal death cause not known as reported at a later date and no information could be obtained.

Other SAEs (non-deaths) include: pneumonia in child, fractured leg in newborn, transverse lie, breech presentation leading to C-section, obstructed labour, omphalocoele, hypotonic uterus, cervical incompetence, puerperal sepsis and mastitis. vanishing twin syndrome

B2) GAMBIA: Main cause of SAEs by study arm

|  | **Control** |  | **Intervention** | |
| --- | --- | --- | --- | --- |
| **Main cause of SAE** | **N** | **%** | **N** | **%** |
| Abruptio placenta | 7 | (11.29) | 6 | (9.23) |
| Anaemia | 3 | (4.84) | 3 | (4.62) |
| birth asphyxia | 8 | (12.9) | 8 | (12.31) |
| Congenital abnormalities | 0 | (0) | 1 | (1.54) |
| malaria | 1 | (1.61) | 1 | (1.54) |
| malaria and pre-eclampsia/hypertension | 0 | (0) | 1 | (1.54) |
| neonatal sepsis | 5 | (8.06) | 7 | (10.77) |
| placental Previa | 4 | (6.45) | 0 | (0) |
| pre-eclampsia/eclampsia | 5 | (8.06) | 9 | (13.85) |
| prolonged labour | 0 | (0) | 1 | (1.54) |
| spontaneous abortion | 3 | (4.84) | 3 | (4.62) |
| stillbirth | 13 | (20.96) | 17 | (26.16) |
| other | 13 | (20.97) | 8 | (12.31) |
| Total | 62 |  | 65 |  |

C1) BENIN: reported SAEs by main cause and outcome

|  |  |  |  | Outcome |  |  |  |
| --- | --- | --- | --- | --- | --- | --- | --- |
| Main Cause | Abortion | Maternal death | Perinatal death | Preterm | recovered | Stillbirth | Total |
| anaemia | 0 | 0 | 0 | 0 | 1 | 0 | 1 |
| birth asphyxia | 0 | 0 | 1 | 0 | 0 | 0 | 1 |
| breech position | 0 | 0 | 0 | 0 | 2 | 1 | 3 |
| cephalopelvic disproportion and fetal distress | 0 | 0 | 0 | 0 | 1 | 0 | 1 |
| eclampsia | 0 | 0 | 0 | 0 | 2 | 0 | 2 |
| foetal distress | 0 | 0 | 0 | 0 | 4 | 0 | 4 |
| lombopelvic pain and contractions starting | 0 | 0 | 0 | 1 | 0 | 0 | 1 |
| malaria and prematurity and breech | 0 | 0 | 0 | 0 | 1 | 0 | 1 |
| placental abruption | 0 | 0 | 0 | 0 | 0 | 1 | 1 |
| premature twins | 0 | 0 | 4 | 0 | 2 | 1 | 7 |
| prematurity | 0 | 0 | 4 | 0 | 1 | 1 | 6 |
| spontaneous abortion | 3 | 0 | 0 | 0 | 0 | 0 | 3 |
| stillbirth | 0 | 0 | 0 | 0 | 0 | 6 | 6 |
| umbilical cord prolapse | 0 | 0 | 0 | 0 | 2 | 0 | 2 |
| vascular renal syndrome | 0 | 1 | 0 | 0 | 0 | 0 | 1 |
| Total | 3 | 1 | 9 | 1 | 16 | 10 | 40 |

C2) BENIN: Main cause of SAEs by study arm

|  | **Control** |  | **Intervention** | |
| --- | --- | --- | --- | --- |
| **Main cause of SAE** | **N** | **%** | **N** | **%** |
| anaemia | 1 | (5) | 0 | (0) |
| birth asphyxia | 1 | (5) | 0 | (0) |
| breech position | 2 | (10) | 1 | (5) |
| cephalopelvic disproportion and fetal distress | 1 | (5) | 0 | (0) |
| eclampsia | 0 | (0) | 2 | (10) |
| foetal distress | 1 | (5) | 3 | (15) |
| lombopelvic pain and contractions starting | 0 | (0) | 1 | (5) |
| malaria and prematurity and breech | 1 | (5) | 0 | (0) |
| placental abruption | 1 | (5) | 0 | (0) |
| premature twins | 4 | (20) | 3 | (15) |
| prematurity | 1 | (5) | 5 | (25) |
| spontaneous abortion | 2 | (10) | 1 | (5) |
| stillbirth | 2 | (10) | 4 | (20) |
| umbilical cord prolapse | 2 | (10) | 0 | (0) |
| vascular renal syndrome | 1 | (5) | 0 | (0) |
| Total | 20 |  | 20 |  |
